# Progress in HIV-1 Integrase Inhibitors: A Review of their Chemical Structure Diversity

**Published:** 2016

**Authors:** Zahra Hajimahdi, Afshin Zarghi

**Affiliations:** Department of Pharmaceutical Chemistry, School of Pharmacy, Shahid Beheshti University of Medical Sciences, Tehran, Iran.

**Keywords:** HIV-1, Integrase enzyme, SAR, Molecular diversity, IN Inhibitors

## Abstract

HIV-1 integrase (IN) enzyme, one of the three main enzymes of HIV-1, catalyzed the insertion of the viral DNA into the genome of host cells. Because of the lack of its homologue in human cells and its essential role in HIV-1 replication, IN inhibition represents an attractive therapeutic target for HIV-1 treatment. Since identification of IN as a promising therapeutic target, a major progress has been made, which has facilitated and led to the approval of three drugs. This review focused on the structural features of the most important IN inhibitors and categorized them structurally in 10 scaffolds. We also briefly discussed the structural and functional properties of HIV-1 IN and binding modes of IN inhibitors. The SAR analysis of the known IN inhibitors provides some useful clues to the possible future discovery of novel IN inhibitors.

## Introduction

The acquired immunodeficiency syndrome (AIDS), caused by HIV-1, has developed into a major global epidemic. According to the latest statistical data, there are about 35.3 million people living with HIV-1 today (1). AIDS and HIV-1 infection have been considered as a serious health threats for humans with magnificent social, economic, and ethical consequences. since it was first reported in 1981, there have been scientific efforts to find good therapeutic approaches to fight this disease. Azidothymidine (AZT) was approved on March 1987 as a first clinically effective anti-HIV-1 drug (2). Emerging of resistance to AZT showed that resistance could be minimized by combining drugs together in a highly active antiretroviral therapy (HAART). HAART comprising three or more different drugs effectively suppresses HIV-1 replication and dramatically has changed AIDS from a lethal disease to a chronic infection (3). Drugs used in HAART target various steps in the HIV-1 replication cycle: entry, fusion, reverse transcription, integration, and protein maturation. The FDA-approved drugs used to treat AIDS classify into four groups: (i) the nucleoside reverse transcriptase inhibitors (NRTI), (ii) the nonnucleoside transcriptase inhibitors (NNRTI), (iii) the protease inhibitors (PIs), and (iv) the fusion inhibitors (4). The efficacy of HAART is slowly declining due to drug resistance conferred by mutations in the HIV-1 genome (5). Thus, there is an urgent need to focus on novel targets. Consequently, new research has resulted in the development of maraviroc as CCR5 antagonist (6) and Raltegravir, the first integrase (IN) inhibitor (7). More recently, two compounds, Elvitegravir and Dolutegravir as IN inhibitors were approved by FDA for the clinical treatment of HIV-1 infections. Among HIV-1 targets, IN is a promising target for the discovery of novel anti-HIV-1 drugs because it has no homologue in the human body. IN inhibitors are consisting of two main classes: integrase strand transfer inhibitors (INSTIs) and protein–protein interaction inhibitors (PPIIs). INSTIs target the enzyme active site, and the FDA-approved IN inhibitors are all INSTIs. IN catalyzes the incorporation of viral DNA into the host chromatin on interactions with various cellular proteins, such as lens epithelium-derived growth factor (LEDGF)/p75. LEDGINs which act as inhibitors of the LEDGF/p75–integrase interaction have been substantially developed in recent years (8-12).

In this review, we provided an insight to the structure and function of HIV-1 IN and its role in HIV-1 replication. We also highlighted progress medicinal chemistry efforts have made to date on IN inhibitors.

*HIV-1 Replication cycle and integrase function*

**Figure 1:**
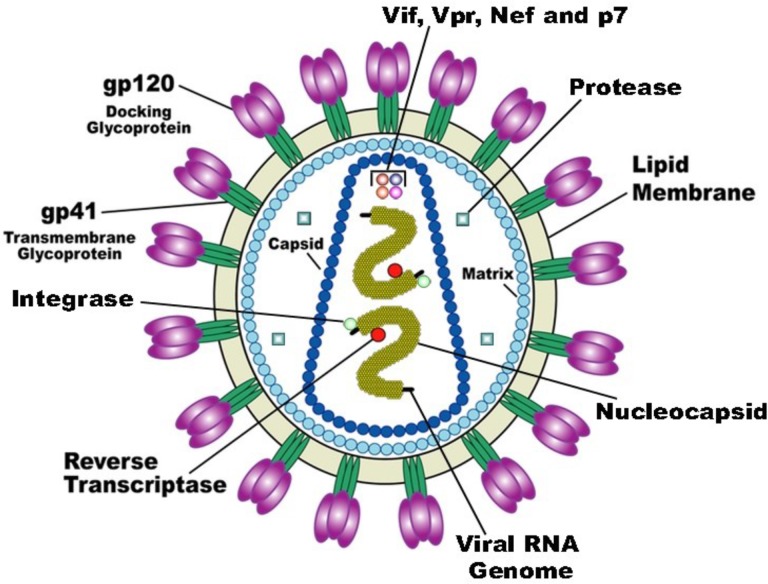
HIV-1 virion structure.

**Figure 2:**
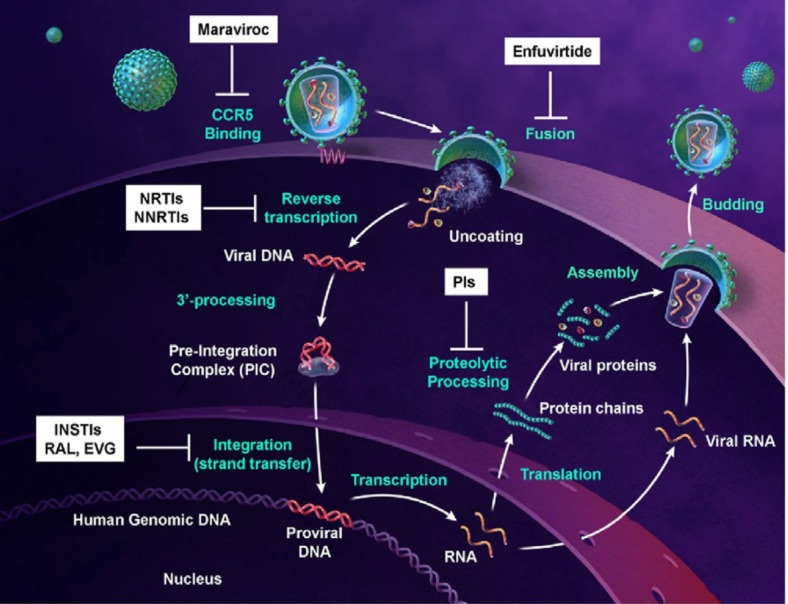
HIV-1 retroviral replication cycle (derived from 22).

HIV-1 is a retrovirus from the lentivirus genus. The viral particle consists of a capsid surrounded by the virion envelope and two copies of the RNA within the capsid core (Figure 1). The RNA sequences are approximately 9 kb in length and code structural (*env*), nonstructural (*gag-pol*), and accessory proteins (Nef, Rev, Tat, Vif, Vpr, and Vpu) (13). The *gag* gene encodes viral proteins p17, p24, and p7/p9 while the *pol* gene encodes three viral enzymes reverse transcriptase, integrase and protease. The HIV-1 life cycle commences by an interaction of infectious virion with the host T-cell membrane receptor molecule CD4 via the viral gp120 surface protein. This interaction initiates a conformational change in the gp120 opening up a site to bind to the chemokine coreceptor CXCR4 or CCR5. This is then followed by fusion of the viral and cell membranes and entry into the cell. After uncoating the viral capsid and release of the viral core into the cytoplasm, the viral RNA is transcribed to viral double-stranded DNA via an RNA dependent DNA polymerization process by reverse transcriptase. At this point in the HIV-1 life cycle, viral DNA is incorporated into the host chromosomal DNA sequence through integrase enzyme. Then, the viral DNA undergoes transcription and translation into viral proteins using the cells’ machinery. The viral DNA also produces copies of HIV-1 genomic RNA that are packaged into the new virions along with viral *gag-pol* polyprotein. Subsequent to budding from the cell, viral protease cleaves the *gag-pol* polyprotein into new copies of the viral proteins to generate mature and functional virion (Figure 2) (14-16).

**Figure 3:**
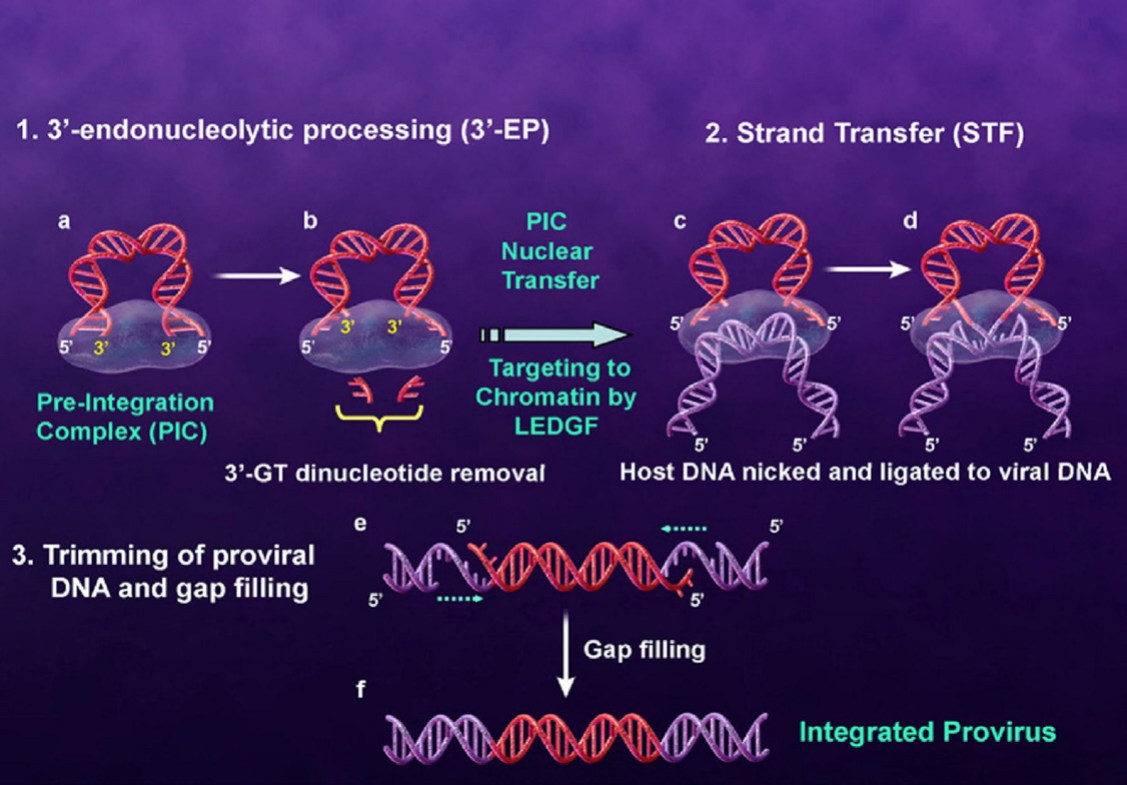
Integration process (derived from 22).

The integrase enzyme which encoded by the 3*ꞌ*-end of the *pol* gene catalyzes the integration of viral DNA into the human genome. The first step of integration process occurs in the cytoplasm where two GT nucleosides from 3*ꞌ*-ends of the viral DNA (that is, long terminal repeat (LTR)) are removed by enzymatic catalysis. In this step which is referred to 3*ꞌ*-processing (3*ꞌ*-P), water attacks the phosphodiester bond as a nucleophile. Integrase bound to the viral DNA as a preintegration complex (PIC). PIC consisting of a variety of viral and cellular factors translocates to the nucleus of the infected cell. In the nucleus, IN binds with the host cell DNA and terminal 3*ꞌ*-OH of the viral DNA attacks the host DNA in a process called strand transfer (ST). Subsequent to ST process two mispaired nucleotides at the 5*ꞌ*-ends of the viral DNA are removed and the strands are fully sealed with the host genome by cell repair enzymes (Figure 3) (17-21). Since integration process is a crucial and unique step in HIV-1 life cycle and IN has no human homolog, so IN is regarded as an interesting drug target (22).

*Integrase structure and mechanism of action*

**Figure 4:**
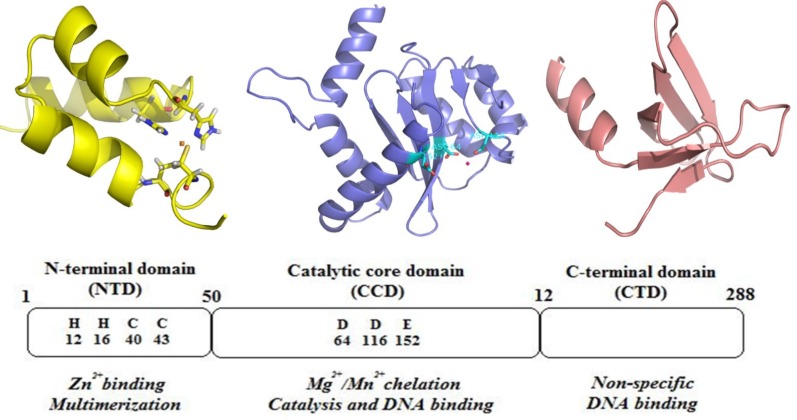
Structural domains of integrase.

HIV-1 IN is a 32 kDa protein belonging to polynucleotidyl transferases family. IN consists of 288 amino acids, and can be divided into three structural and functional domains: a zinc-binding N-terminal domain, a catalytic core domain and a DNA-binding C-terminal domain (Figure 4). N-terminal domain (NTD) (residues 1−50) contains a zinc-binding HHCC motif found to promote tetramer formation which is the active form of the enzyme (23). 

The C-terminal domain (CTD) composes of amino acids 213–288 binds DNA nonspecifically (24). The catalytic core domain (CCD) (residues 50–212) contains a highly conserved catalytic triad of DD(35)E motif, comprising residues Asp 64, Asp116, and Glu152 which coordinates divalent metal ions (Mg^2+^ or Mn^2+^) (25). It has been found that mutagenesis of these residues completely diminishes all catalytic activities (26). These divalent metal ions play a key role in the phosphodiester cleavage and bond formation reactions that are catalyzed during the integration sequence. Some evidences support that these metal cofactors are in fact divalent magnesium ions (27, 28). The CCD contains the enzyme active site, but full catalytic activity requires the N- and C-terminal domains (29).

**Figure 5:**
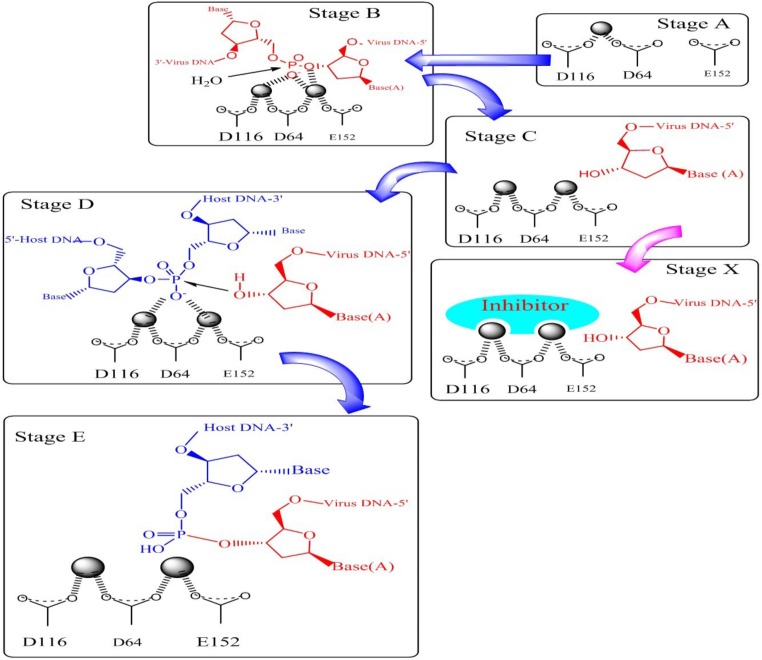
Mechanism of viral DNA integration

Integration process with molecular details is shown in Figure 5. As can be seen from Figure 5. the metal ions stabilize a negative charge of the transition state during phosphate hydrolysis. In 3*ꞌ*-processing step water attacks the phosphodiester linkage and subsequent hydrolysis results in removing of terminal dinucleotide (Figure 5‚ Stage B and C). As shown in Stage D 3*ꞌ*-OH group of the viral DNA serves as a nucleophile and attacks the phosphodiester bond on the host chromosomal DNA and ligates to the 5*ꞌ*-ends of the host DNA (Stage E). It has been demonstrated that sequestration of one or both divalent metal ions by compounds known as two metal binders (Stage X) can lead to the displacement of the viral 3*ꞌ*-OH from the active site, and can prohibit ligation of the viral and host DNA. 

However, a simple metal chelation motif is not sufficient for enzyme inhibition. It has been shown that a hydrophobic aromatic substituent is also required for ST inhibition (30, 31).

**Figure 6:**
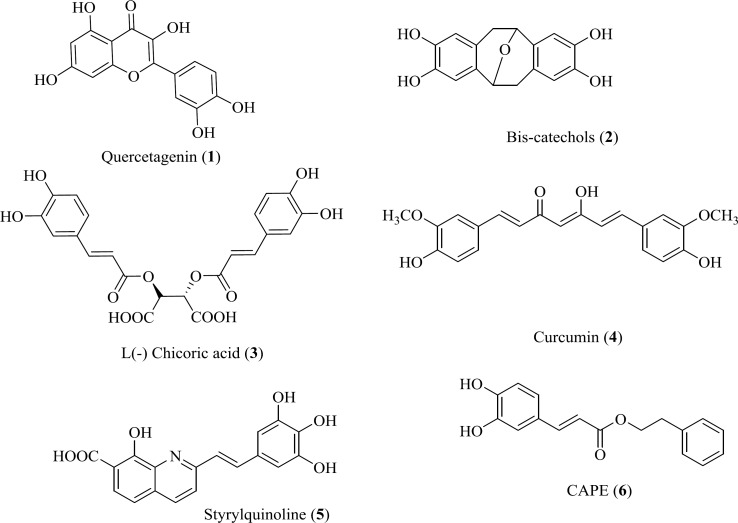
Representative structures of polyhydroxylated aromatic derivatives.

**Figure:**
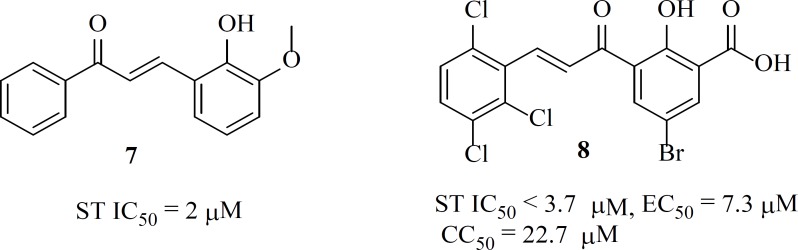


**Figure 7:**
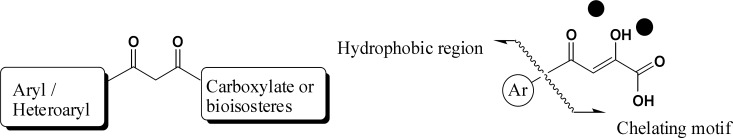
General structures of diketoacids

**Figure 8:**
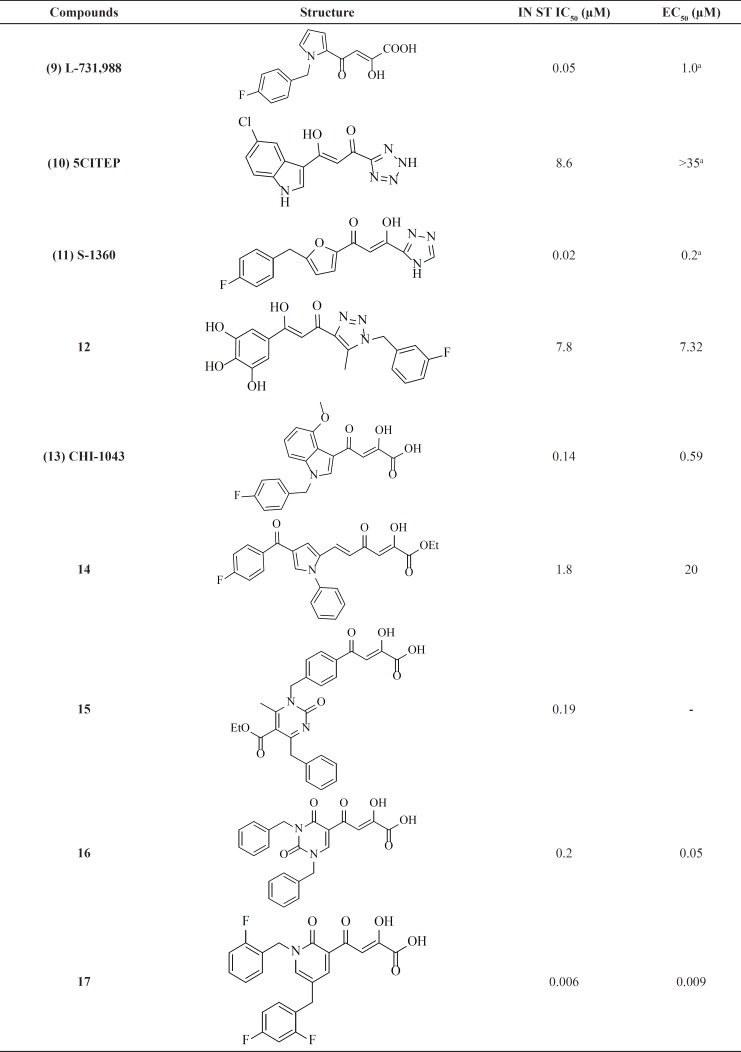
Isosteric replacement of DKA with naphthyridine carboxamide core

**Figure:**
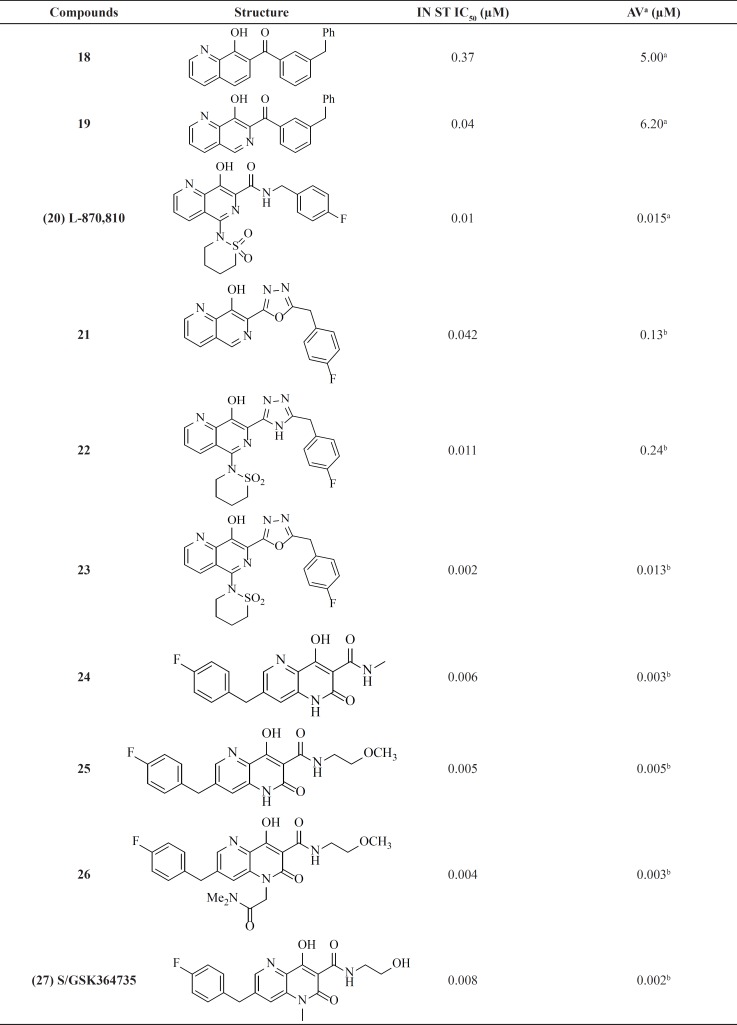


**Figure 9:**
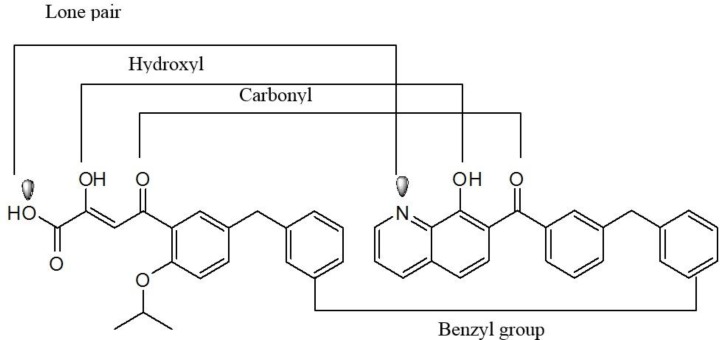
Design of pyrrolloquinolones by cyclizing the L870,810

**Figure 10:**
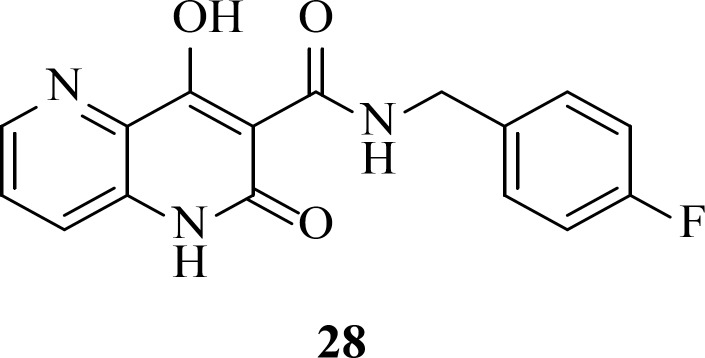
Strategic approach from N-methylpyrimidinones to bicyclic pyrimidinones and pyrido[1,2-*a*]pyrimidin-4-ones

**Figure 11:**
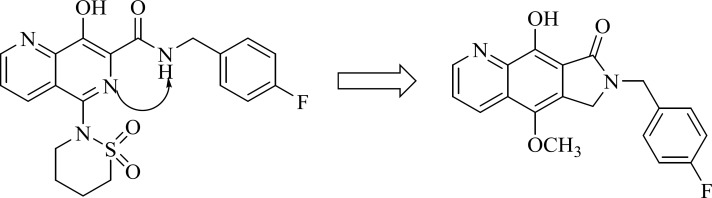
Evolution of N-methylpyrimidone to dihydroxypyrido-pyrazine-1,6-dione

**Figure:**
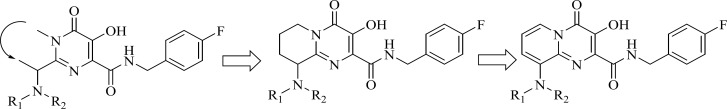


**Figure:**
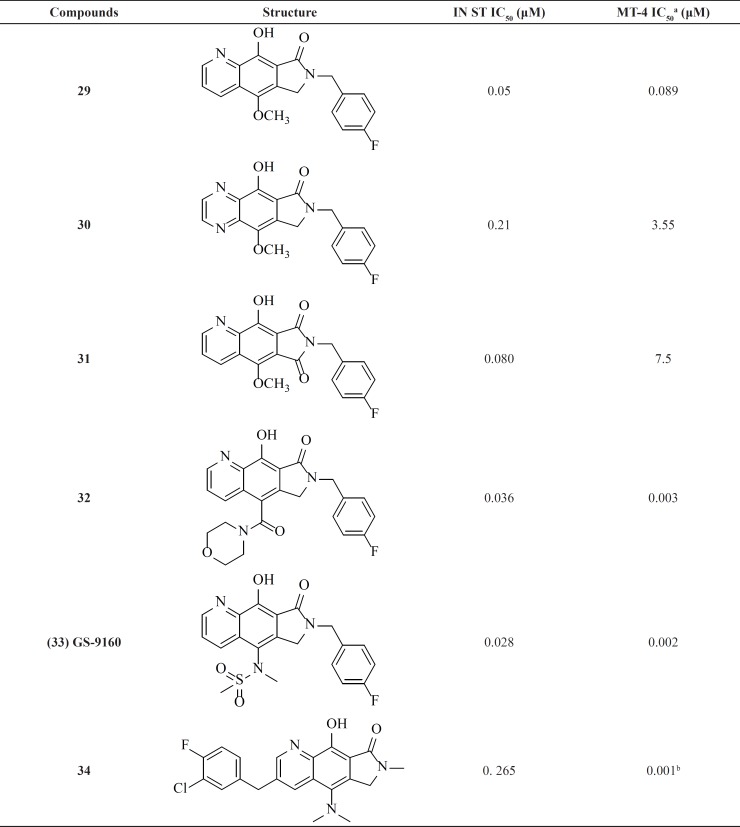


**Figure 12:**
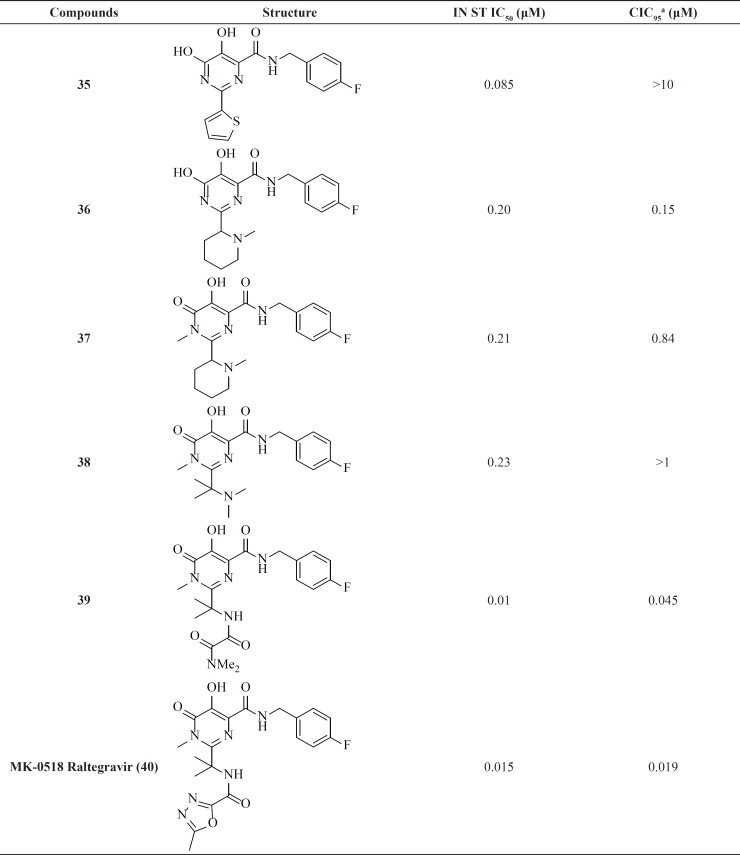
Some quinolone derivatives

**Figure:**
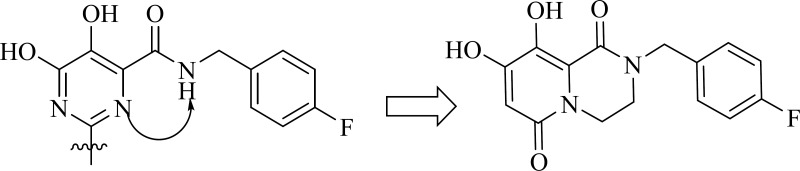


**Figure:**
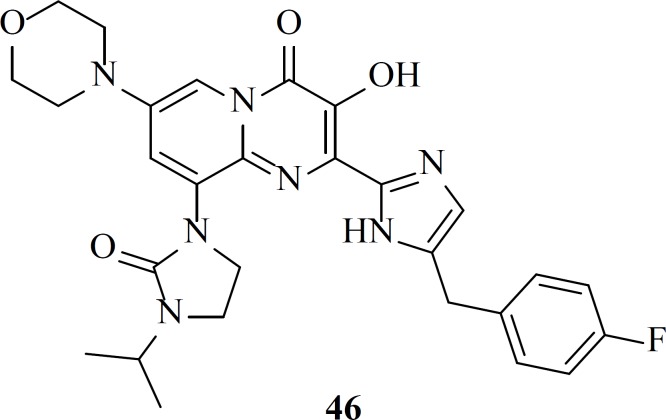


**Figure 13:**
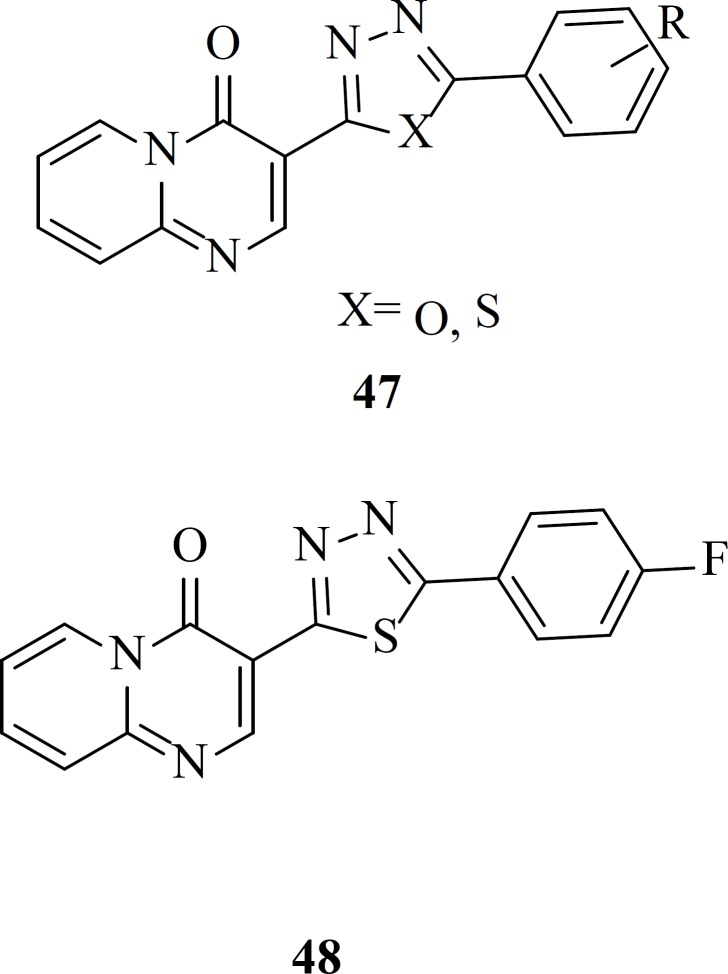
Logical approach toward the cyclization modification of carbamoyl pyridone

**Figure:**
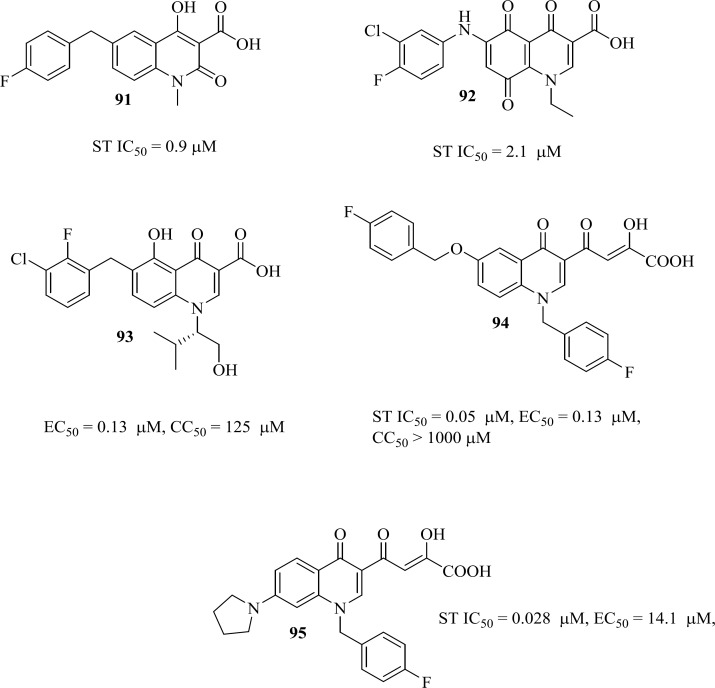


**Figure:**
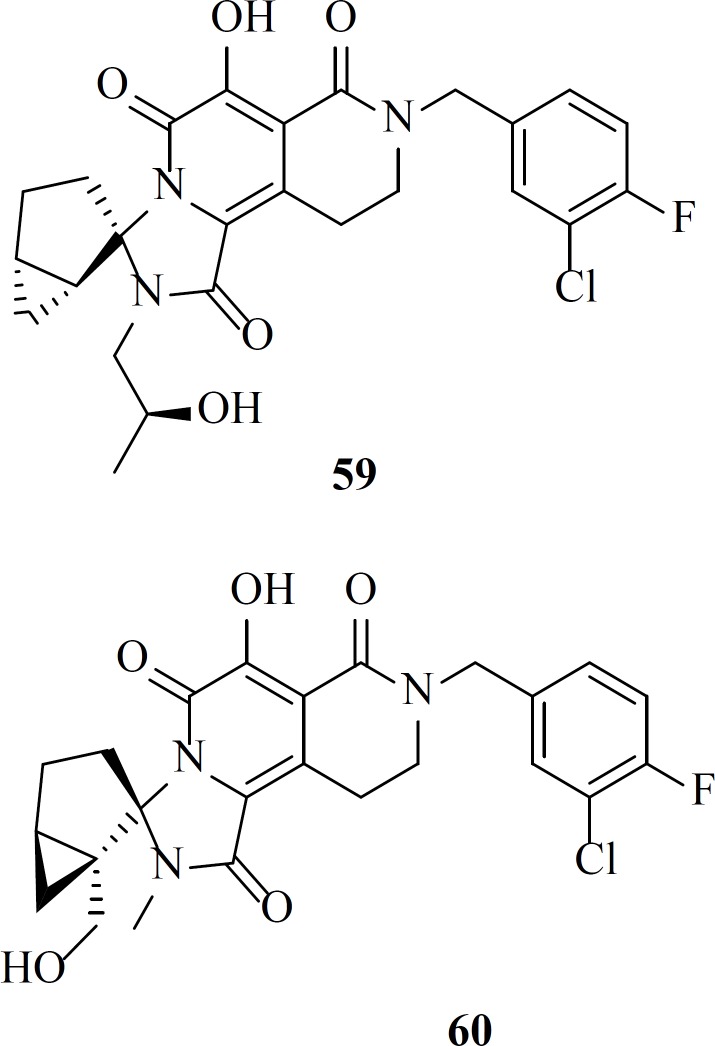


**Figure:**
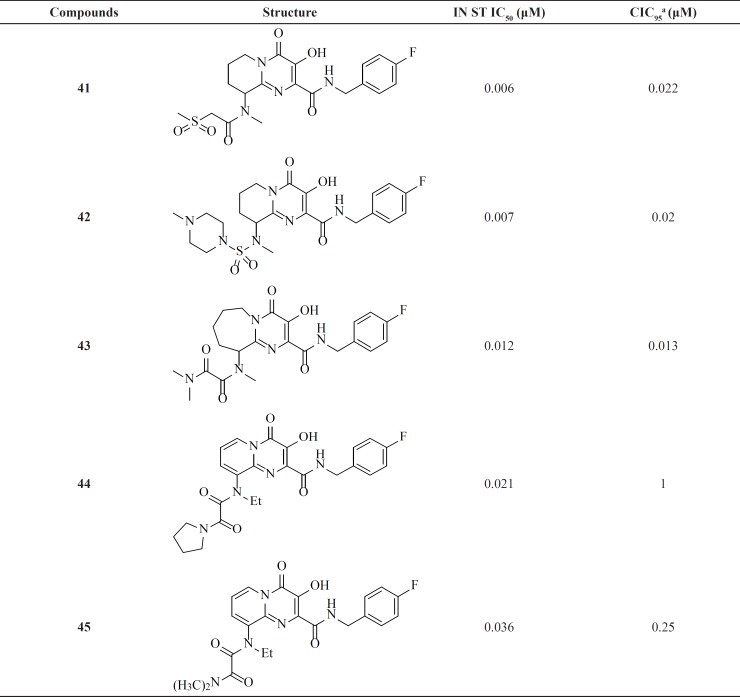


**Figure 14:**
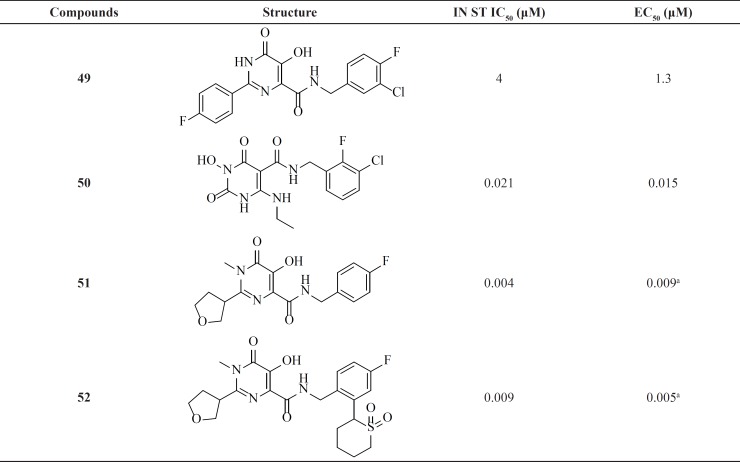
Design of the 8-hydroxyquinoline tetracyclic lactam scaffold

**Figure 15:**
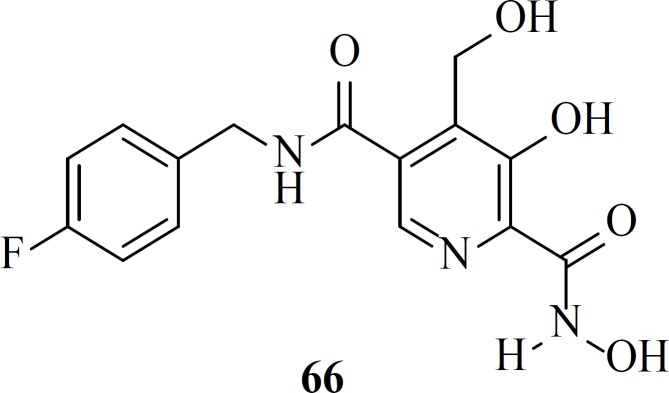
Architecture of the PFV intasome (derived from 32

**Figure 16:**
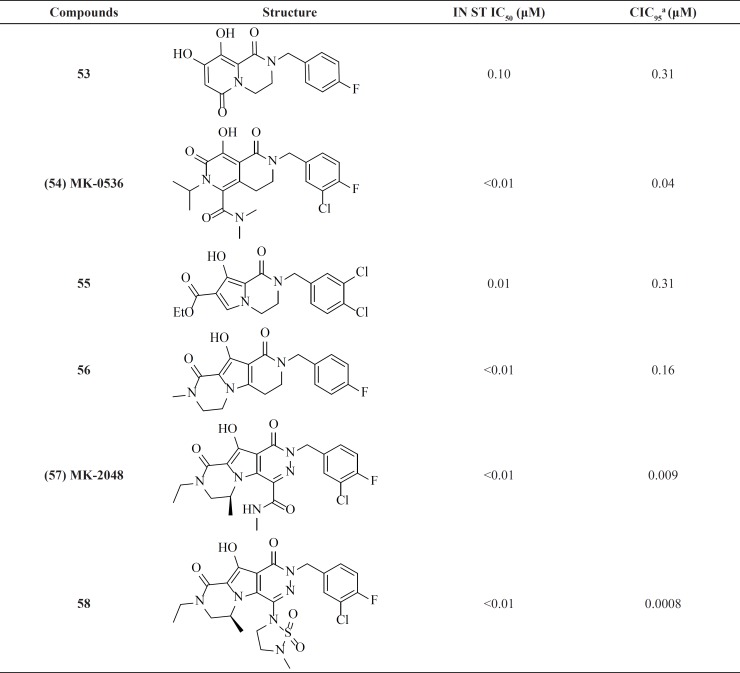
Superimposition of nine drug molecules of various scaffolds in their bound conformations (PDB IDs 3OYA-3OYG and 3L2W) (derived from 124

**Figure 17:**
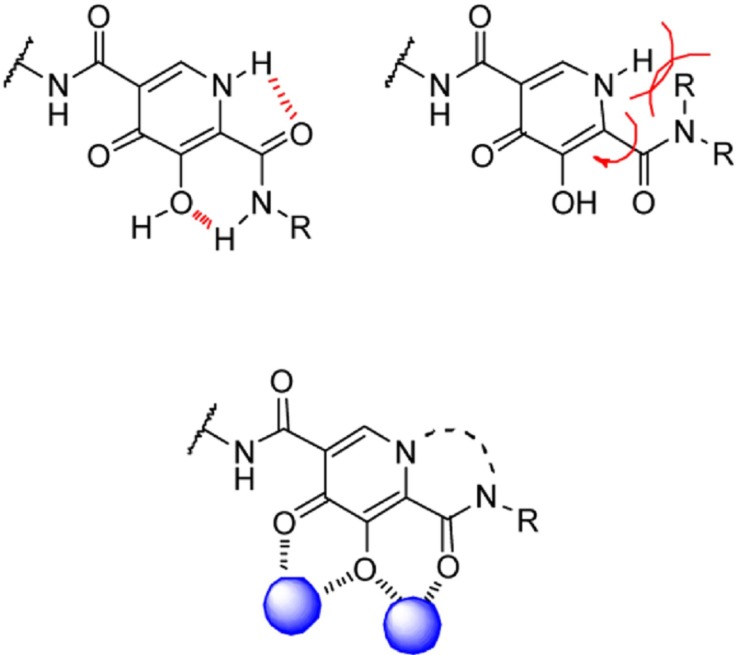
Interactions of Raltegravir within the active site of PFV intasome

**Figure 18:**
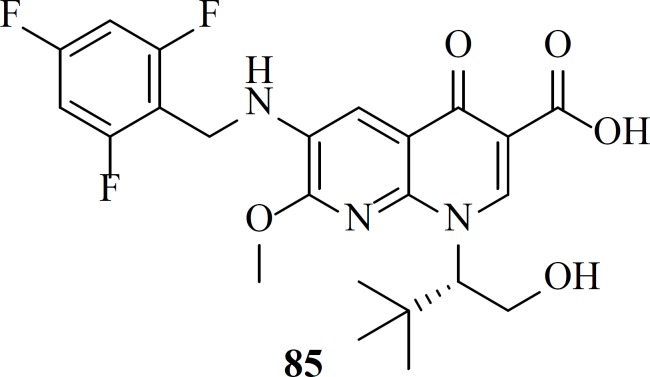
PFV IN active site in committed and drug-bound states (derived from 122

**Figure 19:**
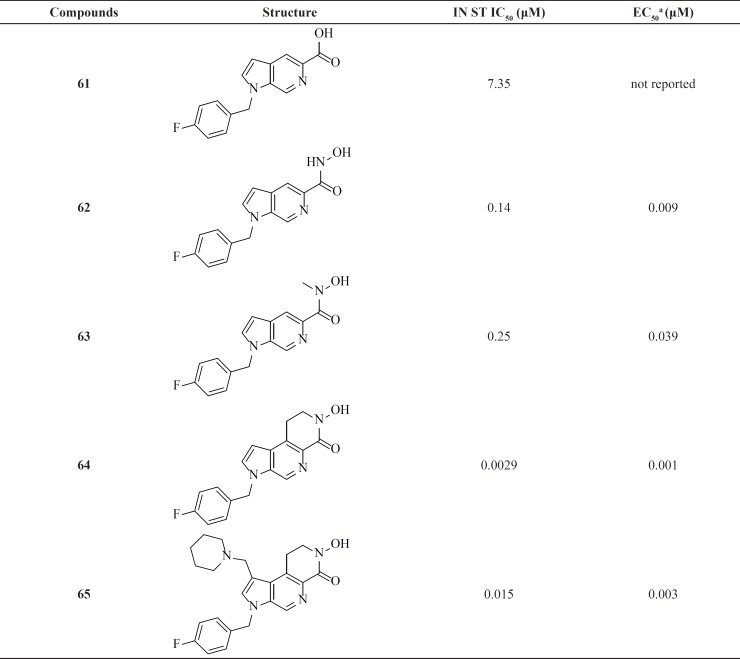
Interactions of Dolutegravir within the active site of PFV intasome

**Figure 20:**
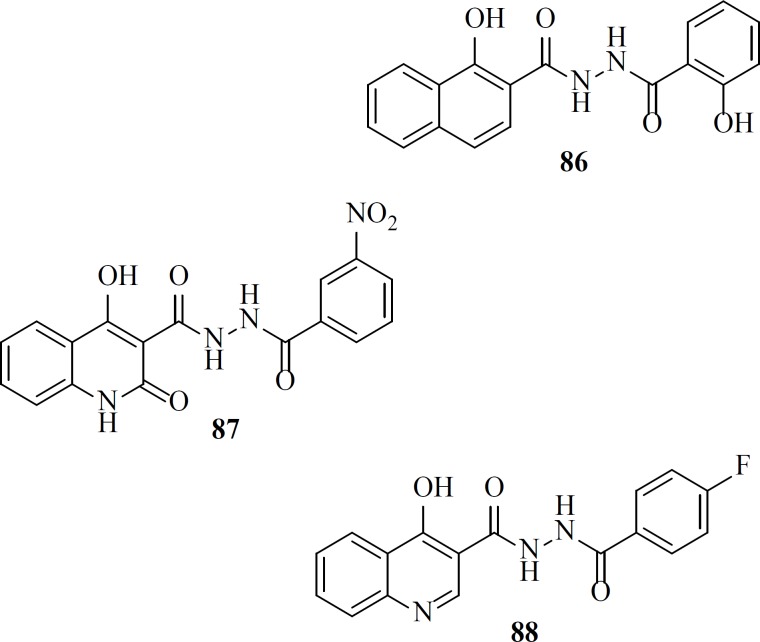
Two-metal binding pharmacophore model for IN Inhibitors

**Figure 21:**
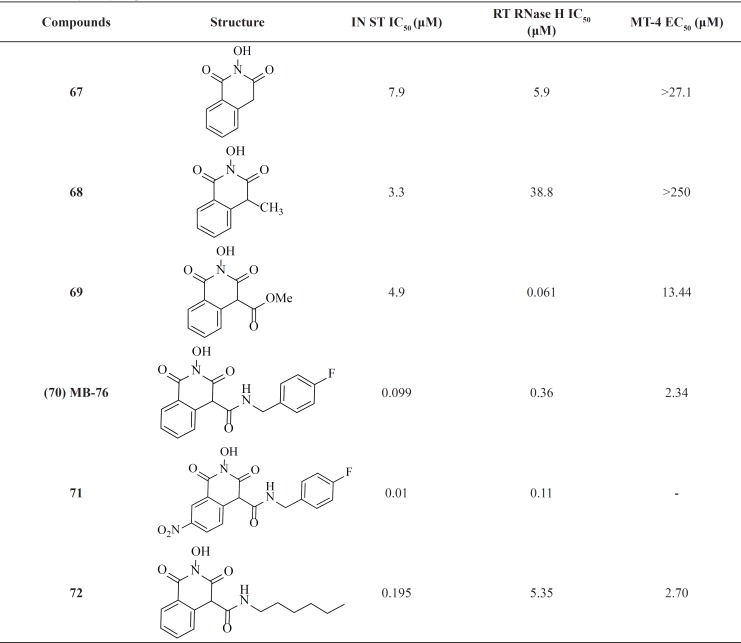
Pharmacophore model of IN inhibitors

**Figure 22:**
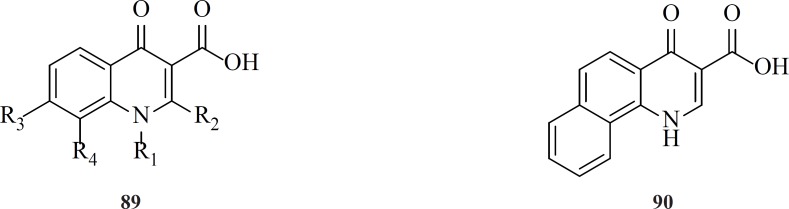
**. **Model templates for metal heteroatom chelation.

**Table 1:** *Diketo acid related derivatives* *.*

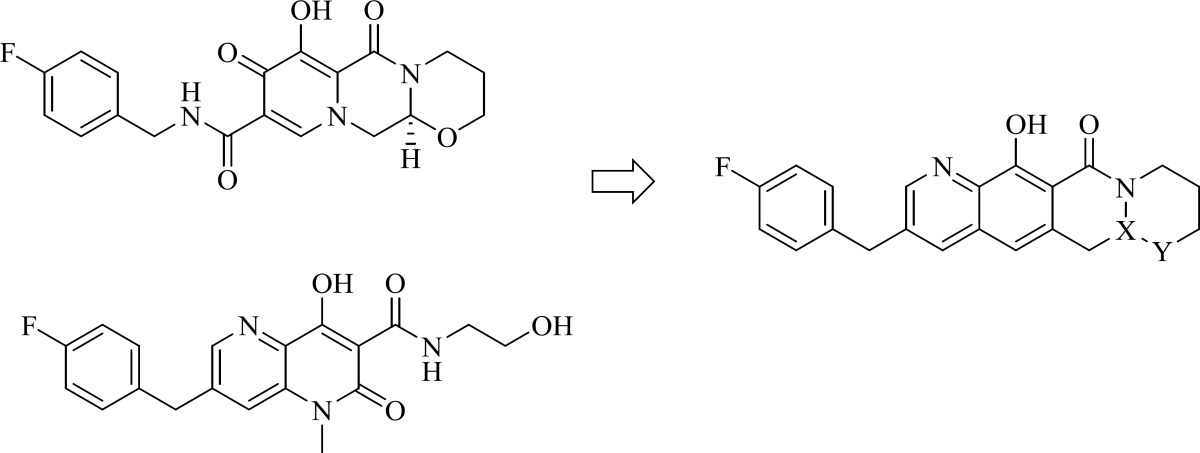

^a^ Antiviral single cycle assay for HIV-1 infectivity IC_50_

**Table 2:** *Naphthyridine carboxamide integrase inhibitors* *.*

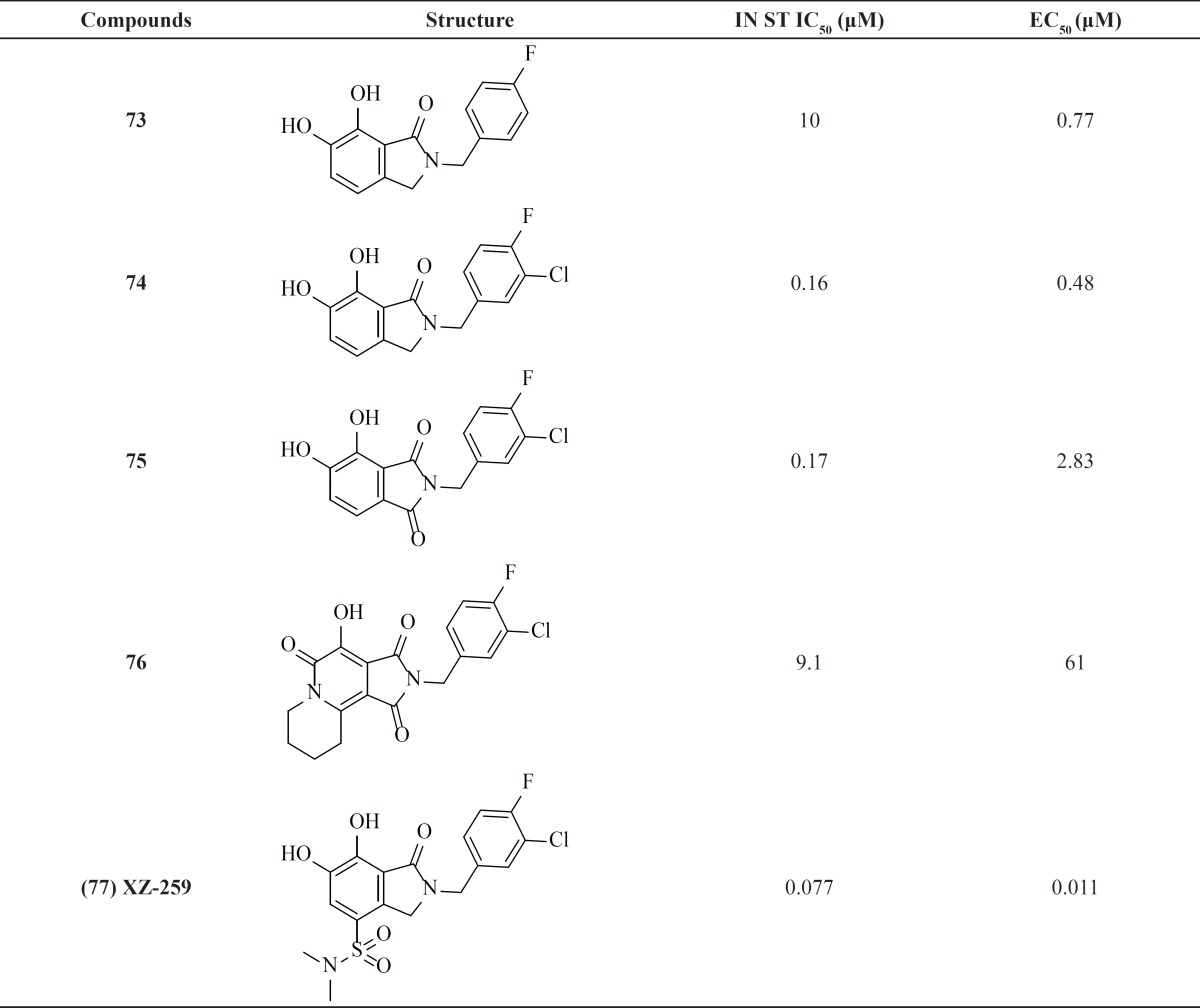

^ a^ Antiviral single cycle assay for HIV-1 infectivity IC_50_

^ b^ Pseudo-typed HIV antiviral assay IC_50_

**Table 3:** Pyrrolloquinolone derivatives

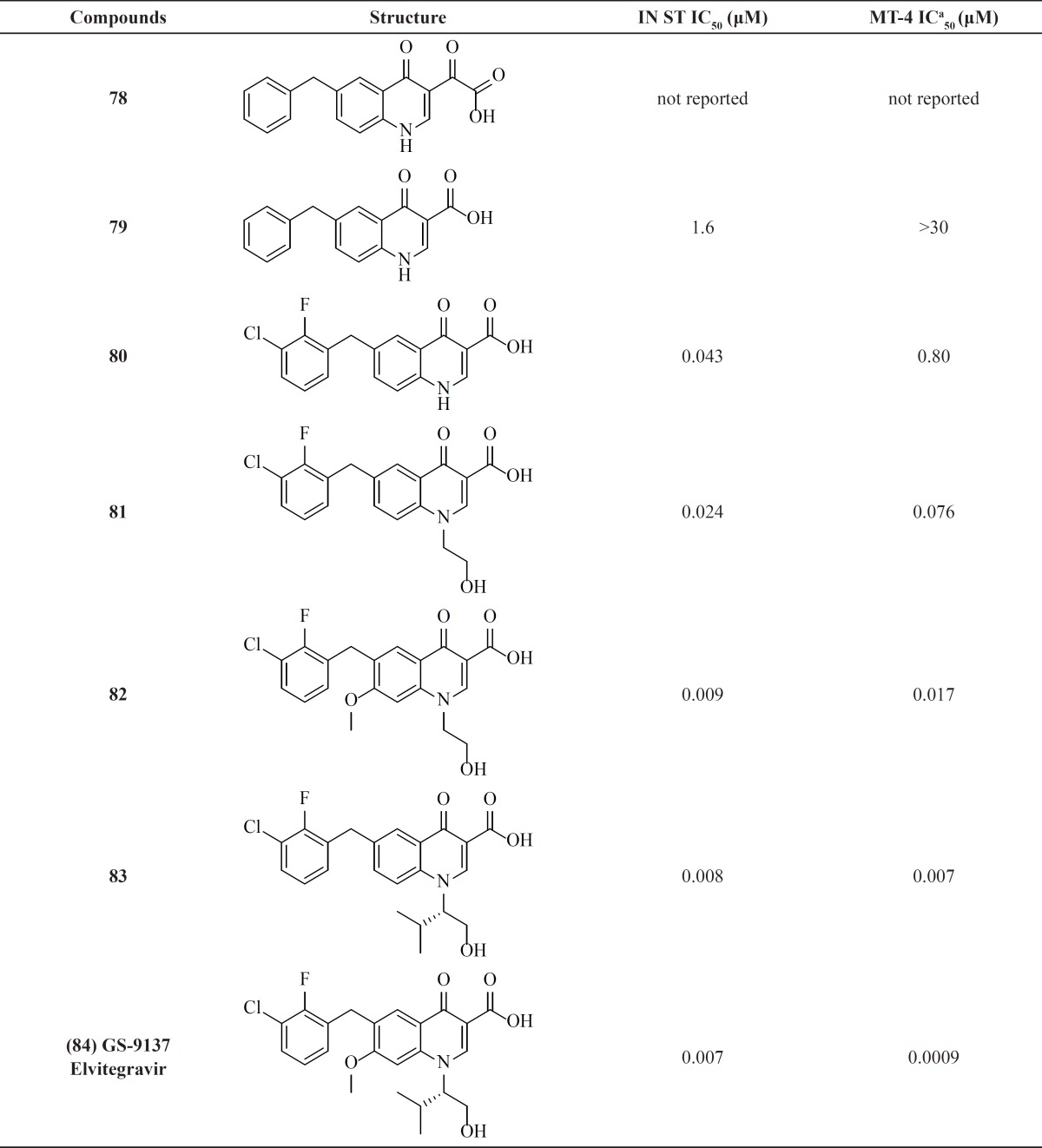

^ a^ MT-4 antiviral assay IC_50_

^ b^ EC_50_ (10% FBS)

**Table 4:** Dihydroxypyrimidine carboxamide derivatives.

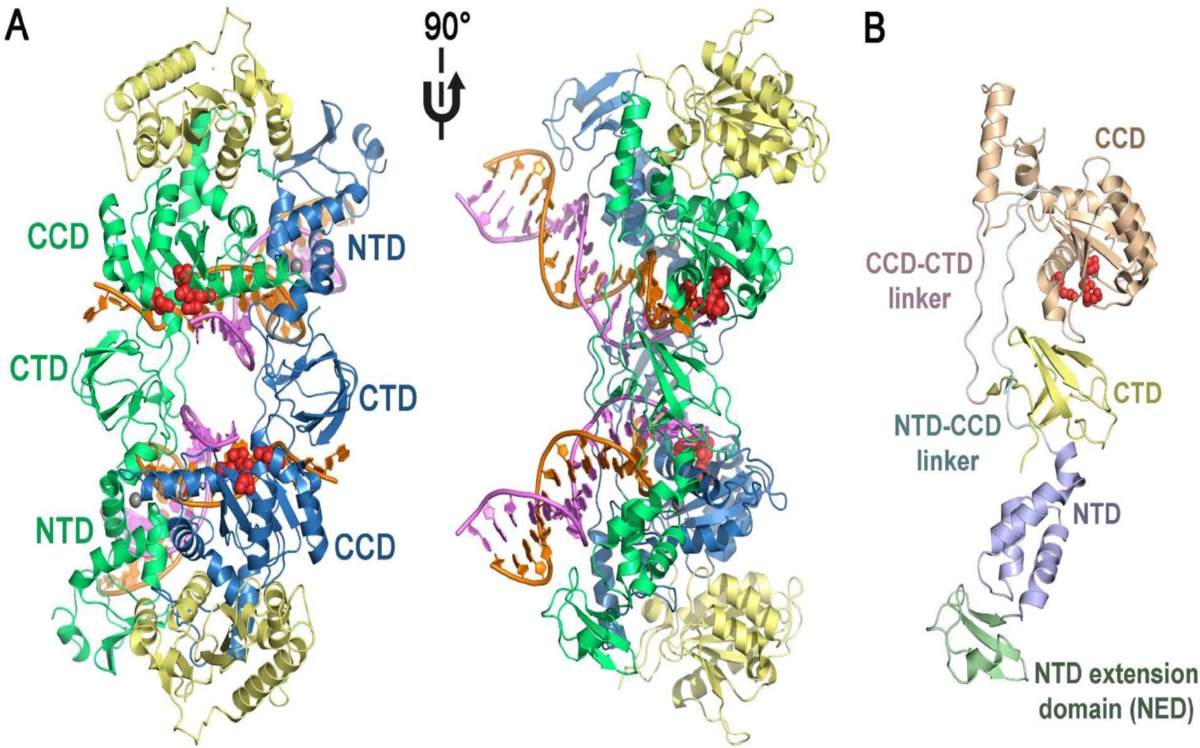

^ a^ Spread multicycle cell-based antiviral assay IC_95_

**Table 5:** Bicyclic pyrimidinones and pyrido[1,2-*a*]pyrimidin-4-ones.

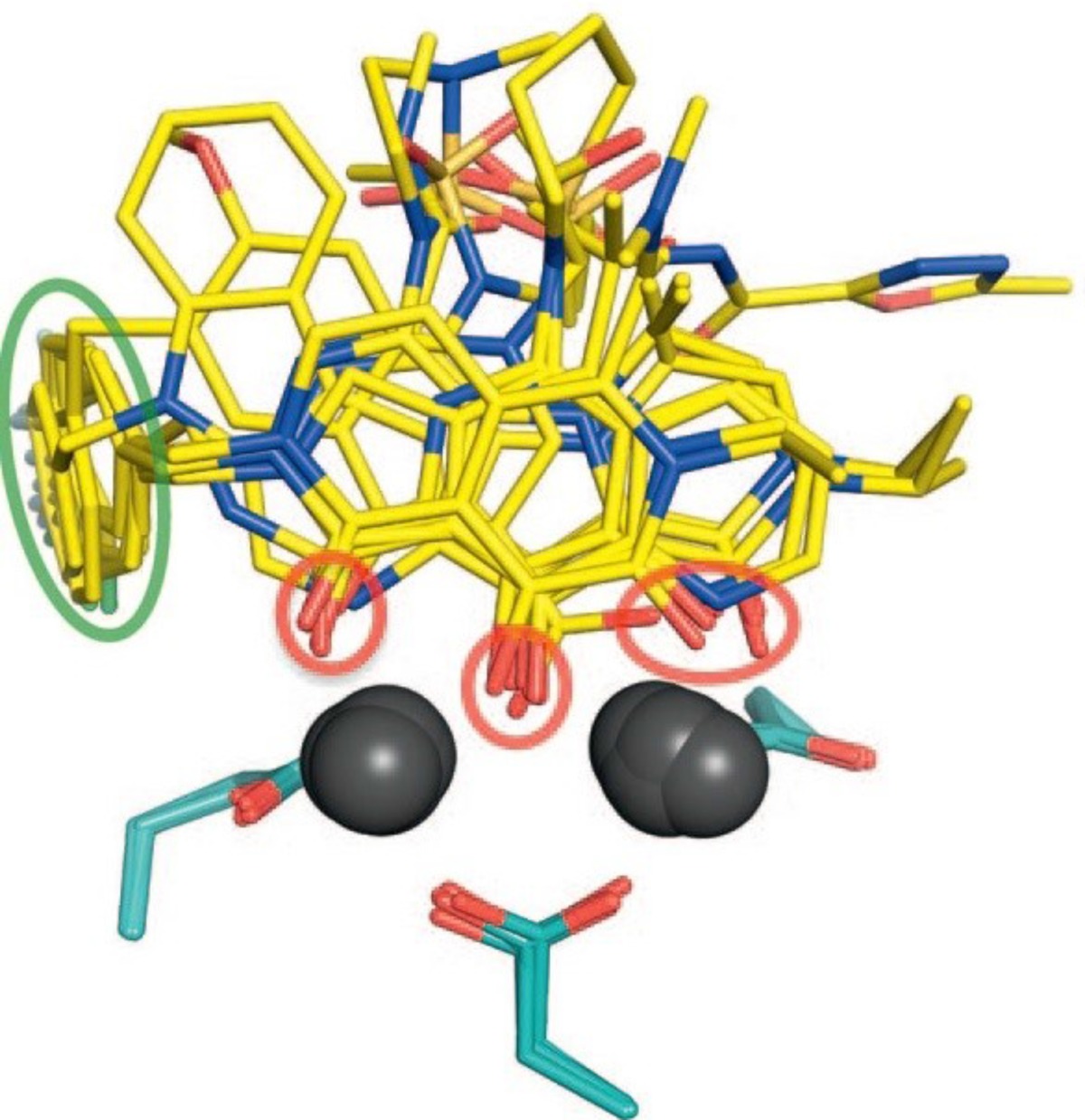

^a^ Spread multicycle cell-based antiviral assay IC_95_ with 50% normal human serum

**Table 6:** Dihydroxypyrimidine derivatives

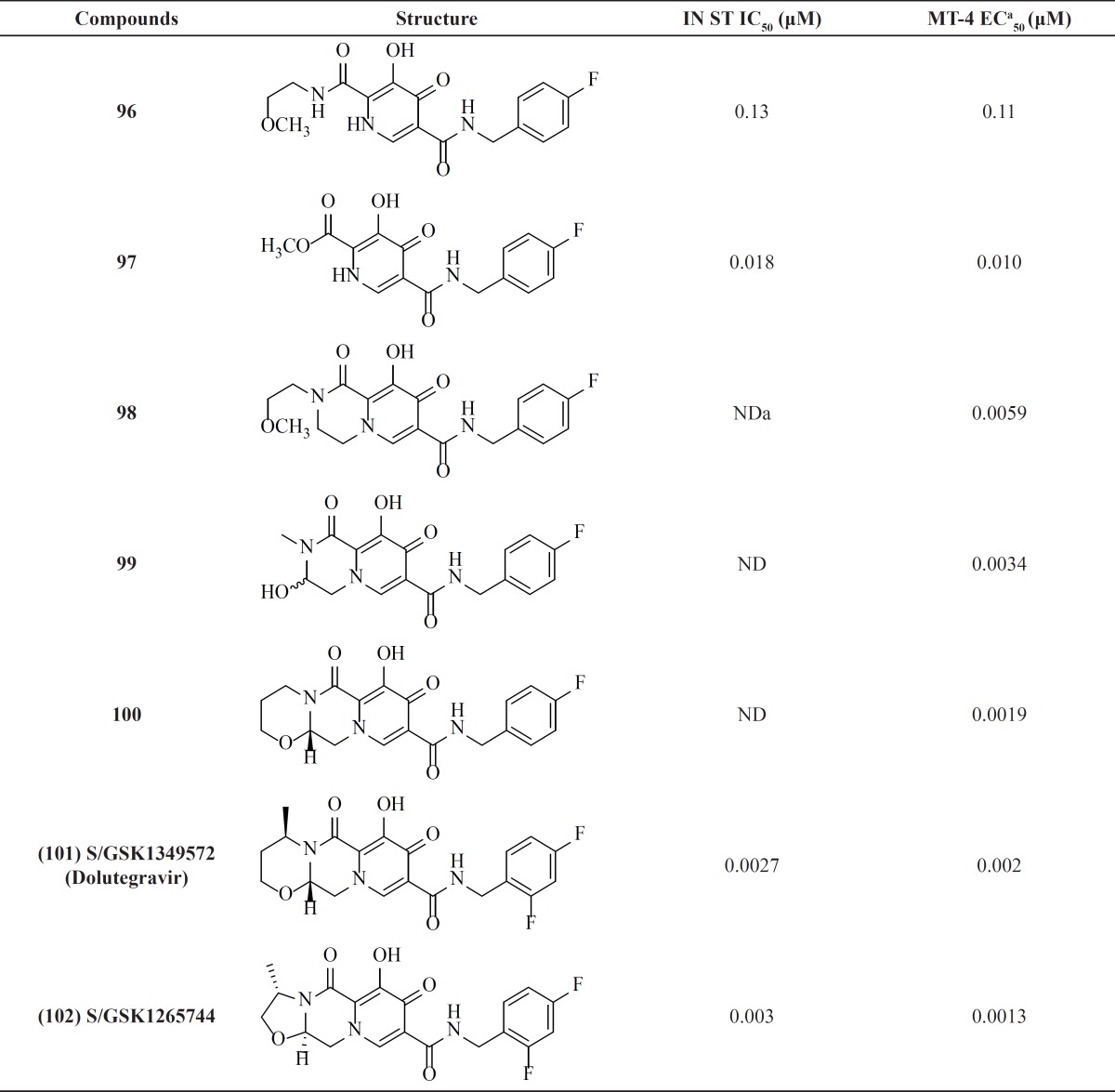

^a^EC_50_ valued were determined in the presence of 10% FBS.

**Table 7:** Dihydroxypyrido-pyrazine-1,6-dione derivatives

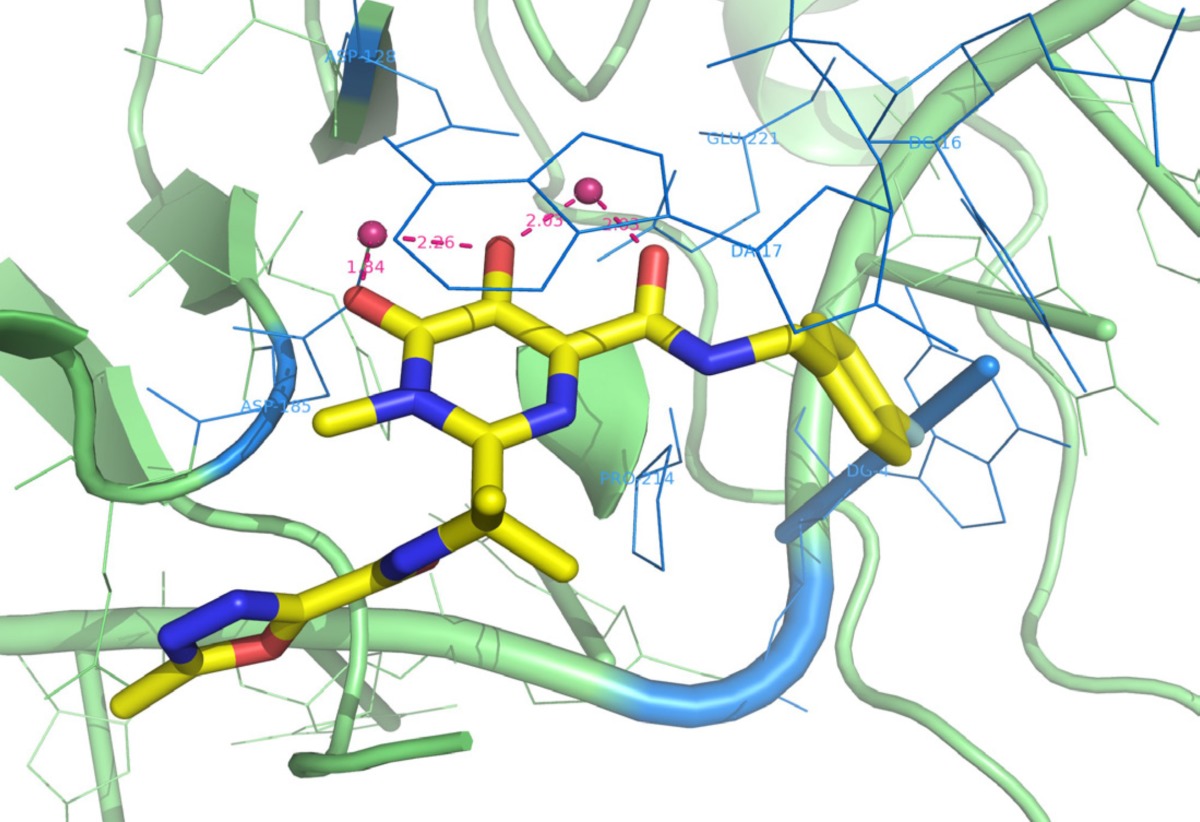

^a^ Spread multicycle cell-based antiviral assay IC_95_ with 50% NHS

**Table 8:** Azaindole hydrixamic acid derivatives

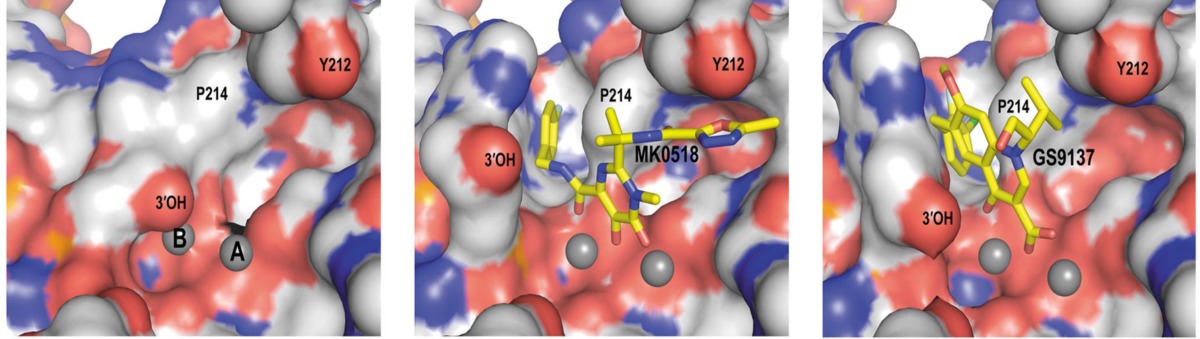

^a^ HIV-1 cytopathic effect (CPE) inhibition assay; EC_50_, 50% effective concentration

**Table 9:** 2-Hydroxyisoquinoline-1,3(*2H,4H*)-dione derivatives

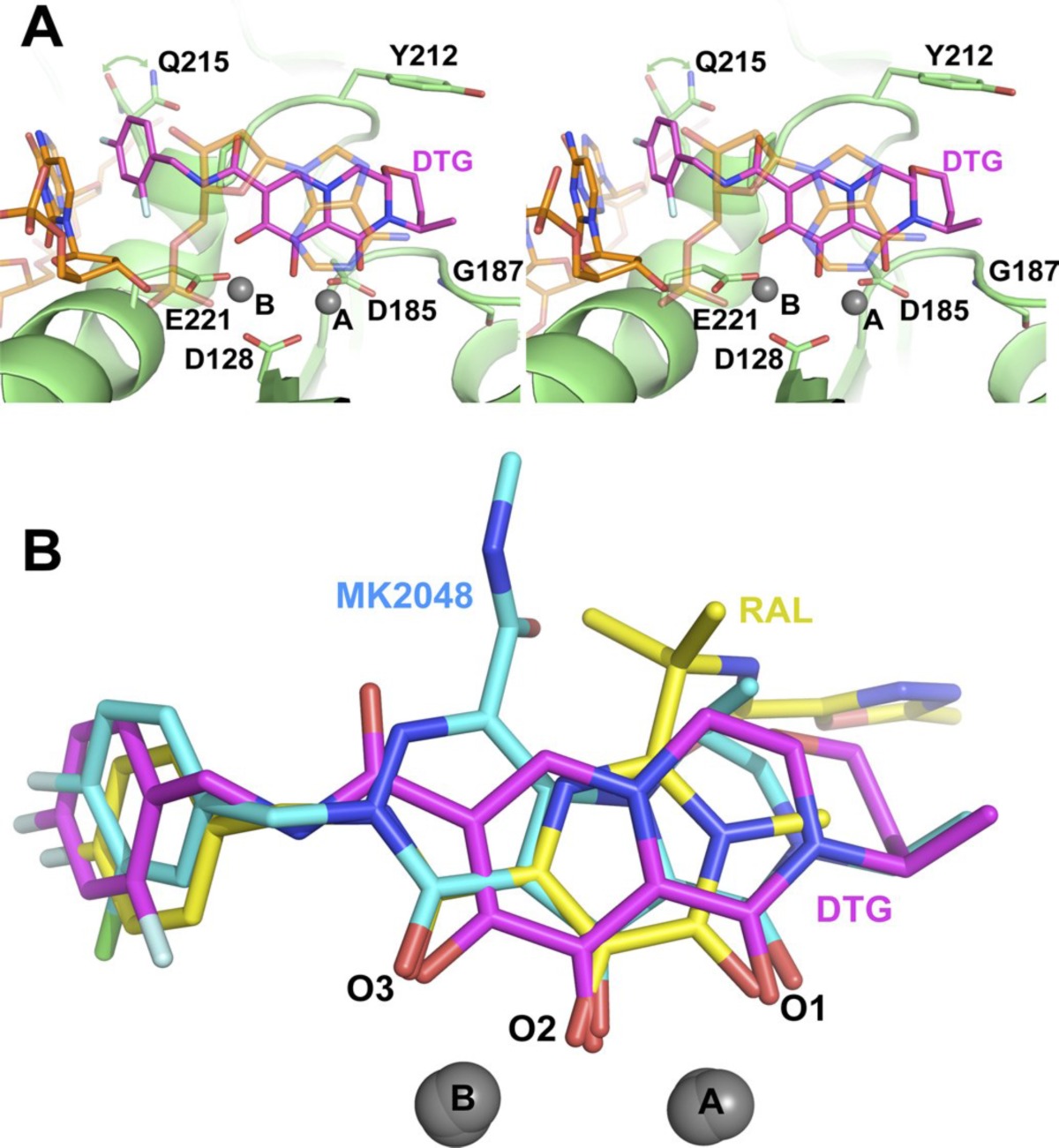

**Table 10:** 6,7-Dihydroxy-1-oxoisoindoline derivatives

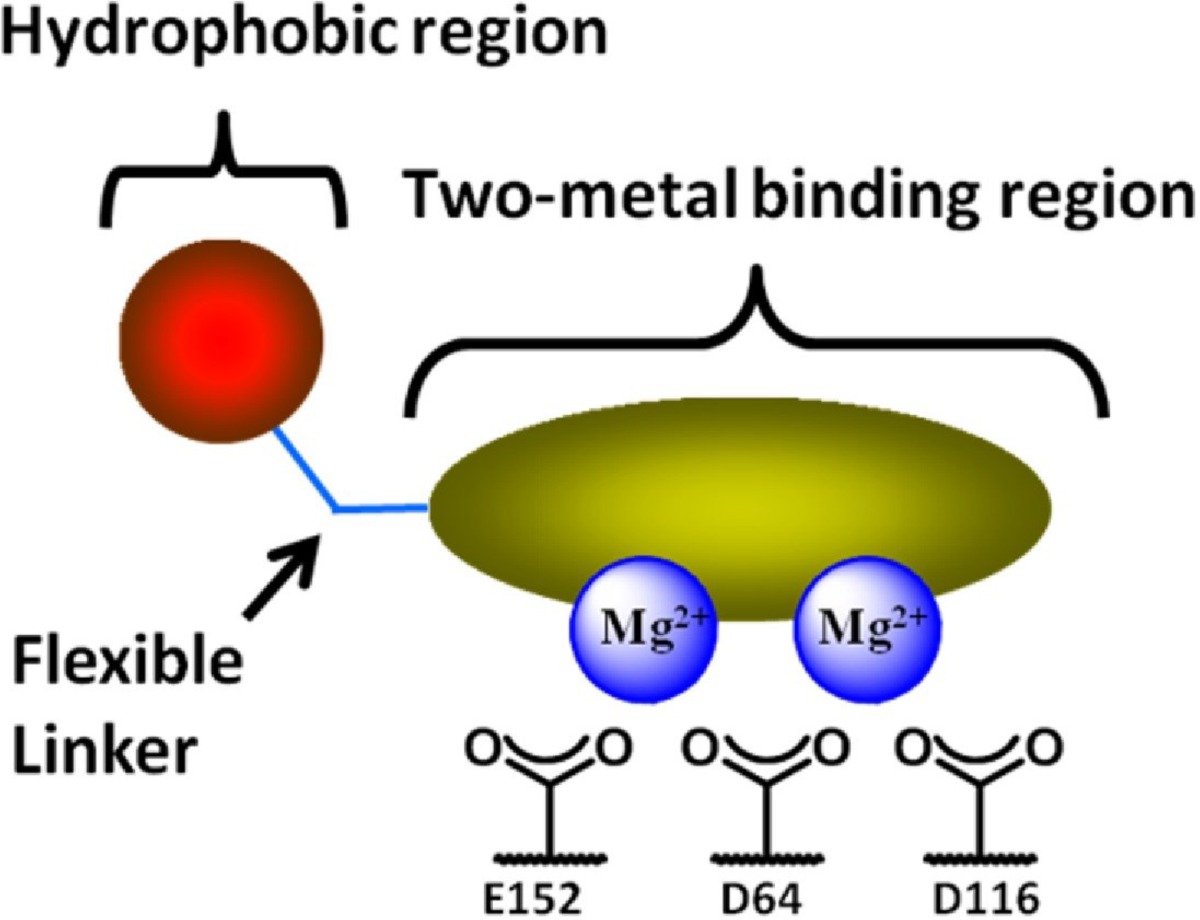

**Table 11:** Quinolone-3-carboxylic acid derivatives

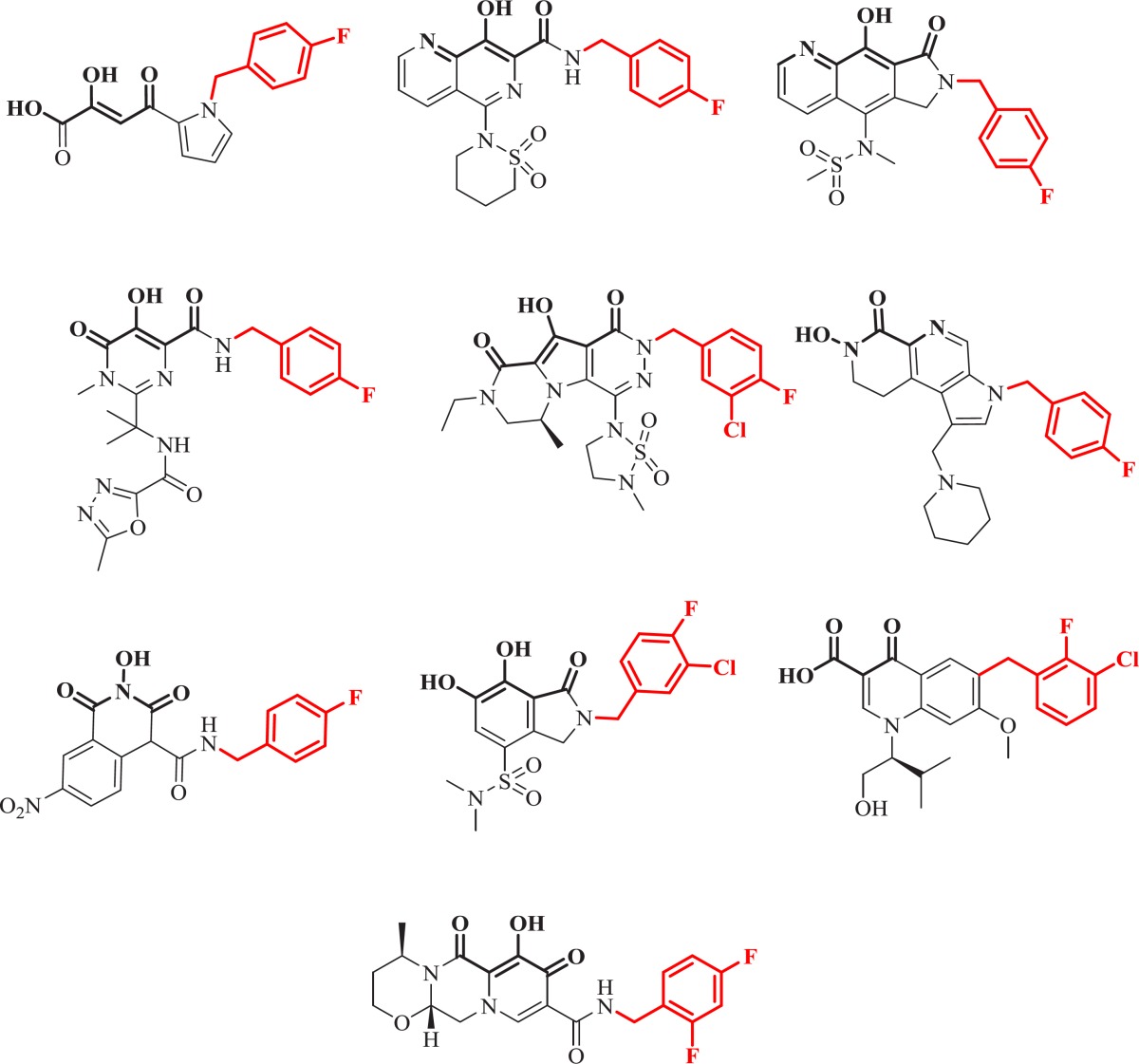

**Table 12:** Carbamoyl pyridone derivatives

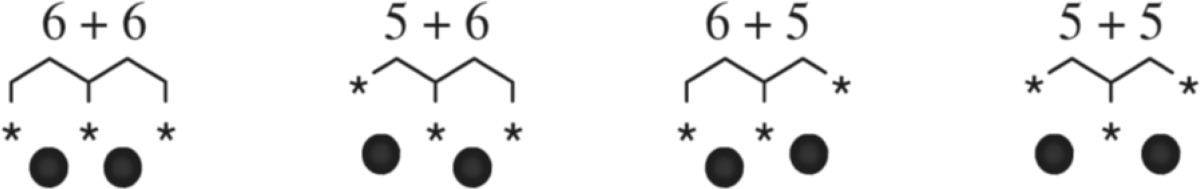

^a^Not Determined

*The discovery of HIV-1 integrase inhibitors*

Early generation of IN inhibitors including peptides, nucleotides, DNA complexes and natural products showed moderate or weak HIV-1 IN inhibitory activities. It was also observed that some compounds possessing inhibitory effect against a recombinant IN did not exhibit antiviral activity in cell-based assays. However, some small molecules were found with a satisfactory inhibitory activity against HIV-1 IN. Therefore, a vast amount of research has focused on the small molecule inhibitors (32). Two decades of tremendous effort have been resulted in promising IN inhibitors. In this review we categorized reported IN inhibitors based on the chemical structures of the compounds. We classified IN inhibitors in 10 scaffolds including hydroxylated aromatics, diketo acids, naphthyridine carboxamides, pyrrolloquinolones, dihydroxypyrimidine carboxamides, azaindole hydrixamic acids, 2-hydroxyisoquinoline-1,3(*2H,4H*)-diones, 6,7-dihydroxy-1-oxoisoindolines, quinolone-3-carboxylic acids and carbamoyl pyridines and provided an insight into their structure activity relationships (SAR).

*Hydroxylated aromatics*

Hydroxylated aromatic derivatives represents one of the largest class of IN inhibitors (33-36). The common structure of this class consists of two aryl groups, one of which contains the 1,2-catechol structure, separated by a linker. Some natural compounds are belonged to this class (Figure 6) (37-40). Structure–activity analysis on this class demonstrated that the IN inhibitory activity is dependent on the catechol moiety (41, 42). There are no definitive conclusions regarding their mechanism of actions. It is ambiguous if the hydroxyl groups chelate magnesium ions or if they simply interact with magnesium ions by hydrogen bonding within the catalytic core. Moreover, other mechanism including redox reaction with thiol groups, intercalation in the DNA substrate and interaction with the viral envelope protein gp120 have been reported in the literature (43). Up to now none of them is suitable for clinical development due to cytotoxic problems.

Some chalcone derivatives (e.g. **7 **and **8**) were identified as IN inhibitors by means of pharmacophore technologies based on lead molecules. The most potent compound displayed an IC_50_ value of 2 µM for both IN-mediated 3*ꞌ*-processing and strand transfer reactions. However, these compounds did not have specificity and showed cytotoxicity (44).

*The birth of the diketo acids*

The first inhibitors of the IN enzyme that were also active in cell replication assays were reported by researchers at Merck and Shionogi in 2000. Random screen of over 250,000 compounds resulted in potent inhibitors containing a distinct β-diketo acid (DKA) moiety that was capable of coordinating metal ions within the IN active site (Figure 7) (45) The active compounds (for example, L-731,988, **9**) from this screen exhibited lower IC_50_ value for strand transfer inhibition. It was found that the DKA containing compounds bind with a 1000-fold higher affinity to IN in complex with 3*ꞌ*-processed viral DNA than to non-complexed IN (10–20 μM versus 100 nM) (46). The variation from enzyme and assays to cellular assays confirmed that diketo acids were not acceptable drug candidates due to permeability concerns. Work to optimize the diketo acid series was reported by a different group utilizing a heterocyclic isostere of the carboxylic acid group as part of the metal coordinating motif (31). The study led to both the first inhibitor co-crystallized with IN (5CITEP, **10**) and the first clinically tested inhibitor (S-1360, **11**) (Table 1). X-ray crystallography displayed that 5CITEP was bound at the active site in the vicinity of the residues Asp64, Asp116, and Glu152 that are known to be involved in metal ions coordination (47). Shionogi’s S-1360, another DKA bioisostere, was the first integrase inhibitor to enter into clinical trials. However, due to a lack of *in vivo* efficacy and pharmacokinetic problems, its development was soon abandoned (48, 49).

Recent years, some new DKA-based IN inhibitors have been reported in the literature (Table 1) (50). Compound CHI-1043 (13), one of the most promising indoles showed anti-HIV-1 activity at submicromolar concentration (51). Another reported compounds (14**, **15) exhibited good potency against IN but were not very potent on HIV-infected cell (52, 53). Some new β-diketo acids (16) were reported with nucleobase scaffolds that showed inhibitory activity against both steps of wild-type HIV-1 integrase and significantly potent *in-vitro *anti-HIV-1 activity (54). In another work some potent diketo acids with pyridinone scaffold (17) were designed and synthesized and exhibited remarkable anti-HIV-1 activity in cell culture (55).

*Naphthyridine carboxamides*

Among numerous attempts to develop integrase inhibitors, The DKAs and their modified counterparts greatly advanced the field as the first IN inhibitors and provided encouraging preclinical data but lacked sufficient potency and exhibited adverse hepatic effects (56). Therefore, researchers from Merck showed that the manipulation of a naphthyridine carboxamide framework instead of DKA moiety conferred similar antiviral activity and strand transfer selectivity (57). Although naphthyridine carboxamide core seems structurally different from DKA compounds, it mimics the ketone, the enole and the carboxyl oxygen, with a coplanar structure (Figure 8).

The most active inhibitor from this class, L870,810 (20), displayed very promising activity and became the second IN inhibitor soon to enter clinical trials (Table 2). The analog L-870,810 was also the first integrase inhibitor to show viral load reduction in HIV-1 infected patients (58). However, clinical evaluation of L-870,810 was halted, due to liver and kidney toxicity after long-term treatment in dogs (59). A group at GlaxoSmithKline replaced benzyl amide with a series of five-membered heterocycles as an alternative metal chelation motif. This replacement resulted in potent enzyme activity clearly showing heterocycles like triazole or oxadiazoles ability to participate in the coordination of two-metal ions. Introduction of a sultam group at C-5 similar to L870,810 significantly improved both enzyme and cellular activities. For example, compound **23** showed antiviral activity of 13 nM (60). Structure-activity analysis of the Merck and GSK series revealed that N-6 of the 1,6-naphthyridine ring system had significant influence on anti HIV activity. It can be concluded that a planar arrangement of the metal binding motif is important for good inhibitory activity. 

Studies on naphthyl-like 6,6-aromatic system resulted in one noteworthy patented naphthyridinone scaffold represented by compound **28**. Limited details about SAR and potency of compound have been reported. However, based on enzymatic and cellular potency naphthyridinone benzyl amide scaffold was proposed to be an acceptable replacement for the related naphthyridine structures like L-870,810 (61).

The naphthyridinone core has been also used to develop next series of inhibitors with an interesting feature (62, 63). In these molecules the hydrophobic 4-fluorobenzyl moiety placed on the opposite end of the metal-chelating fragment. This 180° scaffold reversion allows for an entirely new SAR and substitution possibilities. As shown in Table 2ˏ the series exhibited potent activities against both the enzyme and the cellular system. SAR studies on this series led to the discovery of clinical candidate S/GSK364735, 27. Unfortunately, clinical development of this inhibitor was terminated due to concerns about a limited safety margin from preclinical studies (64).

*Pyrrolloquinolones*

A group from Gilead takes the step to optimize clinically efficacious L870,810 by cyclizing the amide and aromatic core ring system to ensure a coplanar arrangement (Figure 9). Resulted compound with tricyclic scaffold, 29 showed sub-100 nM activity (65). SAR studies revealed that introducing an additional nitrogen led to reduction in activity due to low solubility (compound 30) (Table 3) (66). In this work, the most promising compound was GS-9160, 33 which was taken into phase 1 of clinical trials. Although, it showed a modest to good half-life in preclinical pharmacokinetic studies, it lacked sufficient human PK profile to support once daily dosing (67, 68). 

This group also reported a new series of pyrroloquinoline derivatives that moved the benzyl group of the inhibitor to the C3-position of the inhibitor scaffold. Antiviral and enzyme assays demonstrated that this change to the tricyclic scaffold was well-tolerated. The representative compound 34 also displayed good oral bioavailability (69).

*Dihydroxypyrimidine carboxamides: The milestone of the metal chelation strategy*

Among IN inhibitors, the most noticeable series would have to be the dihydroxypyrimidine carboxamides series that have resulted in first FDA-approved IN inhibitor, Raltegravir. The discovery story of Raltegravir has been reported by Rowley in a review of the joint Merck and IRBM research teams (70). The initial lead of this series was developed through screening of inhibitors of HCV polymerase, which demonstrates a high degree of structural similarity to IN. First IN inhibitors in this series (35) with IN ST IC_50_ of 85 nM was inactive in antiviral assay (Table 4) (71). The thiophene ring in the 2-position of the pyrimidine core was found to have little effect upon the interaction of the compound with IN, so replacement of it with a basic amine (compound 36) resulted in good potency in the cell based assay. N-methyl pyrimidinone series showed lower antiviral potency than their dihydroxy counterparts but had improved PK profile. In these molecules hydroxyl and carbonyl groups involved in metal ions chelation. A further optimization study on N-methyl pyrimidinone series led to discovery of Raltegravir (MK-0518, 40), that was approved in October 2007 for the treatment of HIV-1 infection in combination with existing antiretroviral agents in HAART. In December 2011, it was also approved by FDA for pediatric use (72, 73).

Researcher at Merck and IRBM modified the *N*-methylpyrimidinone structure and converted it to bicyclic pyrimidinone and pyrido[1,2-*a*]pyrimidin-4-one scaffolds as new class of HIV-1 integrase inhibitors (Figure 10). The bicyclic pyrimidinones showed nanomolar activity in the inhibition of HIV-1 infection in cell culture (Table 5). 

Therefore, an extensive SAR study using a wide range of substitutions such as amine, amide, sulfonamide, sulfamide, and ketoamide on the saturated ring of the bicyclic structures was employed. In this work compound 43 was proved to be a very potent and selective IN inhibitor with good pharmacological profile in preclinical species and was taken into further development (74). The pyrido[1,2-*a*]pyrimidin-4-one derivatives characterized by eliminating the presence of any stereocenters in the core of the inhibitor exhibited nanomolar activity both in the enzymatic and in the cellular assay but in higher value in comparison to bicyclic pyrimidinone. 

In this series The PK profile of the representative compound 45 showed good parameters (75). 

A different group modified bicyclic pyrimidinone derivatives by replacing the amide group with azoles as a component of a metal binding motif. Eight different five-member ring azoles were examined and it was found that they can efficiently serve as an amide isostere. Among the studied substitutions compound 46 showed the most potency with an EC_50_ value of 6 nM (76).

Our group reported a new series of pyrido[1,2-*a*]pyrimidine derivatives containing 1,3,4-oxadiazole and 1,3,4-thiadiazole rings (47) as a part of the metal chelation. In drug design heterocycles like oxadiazole ring attract great attention due to their versatile nature and well-known biochemical properties (77). All the target compounds were completely safe and exhibited no cytotoxicity at concentration of 100 µM. The most active compounds in this work, 48 showed 48% inhibition rate against single-cycle replicable HIV NL4-3 virions (78)

Since discovery of Raltegravir (RAL) as the first FDA-approved IN inhibitor, different groups taken the step to modulate its structure to identify new inhibitors. Some RAL-based inhibitors have been reported recently. As can be seen from Table 6 compounds 51 and 52 featuring tetrahydrofuran-3-yl moietiy as optimal C2-substituents demonstrated nanomolar activity in both enzyme and cell culture assays (79-81).

Parallel to the N-methylpyrimidone studies, the same group at Merck developed new dihydroxypyrido-pyrazine-1,6-dione derivatives by cyclizing amide side chain of N-methylpyrimidones (Figure 11). New scaffold featured by coplanarity of the amide carbonyl group in the constrained ring and a resulting limitation of flexibility of the 4-fluorobenzyl side chain which was found to be essential for inhibitory activity.

Compound MK-0536, 54 inhibited IN strand transfer *in vitro* at an IC_50_ of 100 nM and HIV-1 replication in cell culture at a CIC_95_ of 40 nM, with little cytotoxicity (Table 7) (82). replacing pyrido ring with pyrrole associated with cyclization led to new tricyclic compounds 57 and 58 with potent activities against wild-type virus. MK-2048 is now in advanced clinical development (83).

Recently a group described the discovery of a new class of HIV-1 integrase strand transfer inhibitors based on the 2-pyridinone core of MK-0536. Their efforts resulted in the establishment of two lead compounds, 59 and 60 with EC_50_ value of 67 and 32 nM, respectively and preclinical pharmacokinetic profiles (84). 

*Azaindole hydrixamic acids*

A research group at Pfizer identified azaindole carboxylic acids with modest activity (IC_50_ = 0.95-7.35 μM) by screening methods (Table 8). Further lead optimization was employed to improve antiviral activity. Replacement the carboxylic acid by a hydroxamic acid resulted in a 40-fold improvement in potency in the enzymatic assay. Although azaindole hydroxamic acids showed potent antiviral cell-based activities but they were mutagenic. 

Therefore, new scaffolds were designed through cyclization of the N-methyl hydroxamate to the main core, locking the metal binding motif into the bioactive conformation (for example, compounds 64 and 65). This modification conferred a better antiviral potency and improved safety margins (85, 86).

In 2016 Stranix *et al.* utilized hydroxamic acid moiety at the structure of pyridoxine core. SAR study of these molecules (e.g. 66) illustrated that antiviral activity of them was influenced by the aryl substitution and aryl-spacer at the 5-position of the main core. Due to favorable pharmacological data further studies are ongoing on this series (87). 

*Hydroxyisoquinoline-1,3(2H,4H)-diones*

2-Hydroxyisoquinoline-1,3*(2H,4H*)-diones were reported as dual inhibitors of IN and the ribonuclease H (RNase H) function of reverse transcriptase (RT) (88). HIV-1 integrase and the RNase H domain of HIV-1 RT show striking similarities in terms of structure and function. SAR studies revealed that introduction of lipophilic alkyl moieties at position 4 increased IN selectivity (Table 9). Further optimization of this scaffold through utilizing carboxamido chains at position 4 led to compound MB-76, 70 with low micromolar anti-HIV-1 activities (89-91). Introduction of electron-withdrawing functional groups such as the nitro moiety at position 7 was beneficial for antiviral activity since nanomolar anti-HIV-1 activities were obtained for this scaffold (compound 71) (92). Recently, a group investigated the effect of the intoduction of various alkyl chains instead of phenyl and benzyl groups. This replacement resulted in hit compound 72 displaying integrase inhibition in the low nanomolar range. Based on their results optimal length of six carbons for the alkyl side chain can be correlated with the optimal fitting within the hydrophobic pocket of IN. The good biological profile of compound 72 and its high barrier to resistance was encouraging for further pharmacomodulate optimization (93).

*6,7-Dihydroxy-1-oxoisoindolines*

New chelating inhibitors were designed by constraining three oxygen atoms in a coplanar arrangement with 6,7-dihydroxy-1-oxoisoindoline scaffold (94) (Table 10). NIH (National Institutes of Health) research team examined some modification on this scaffolld in order to reduce cytotoxicity problems. SAR study data suggested that in general dihalo-substituted compounds posessed higher potency than monohalo-substituted compounds (95). However, compound 75 was cytotoxic because of the embedded catechol functionality. It was proposed to remove the catechol nature of these inhibitors through introduction of a nitrogen substituent into the aryl ring. Resulting tricyclic derivatives retained other key features for IN inhibition and showed low micromolar potencies. It was found that utilizing a sulfonamide or carboxamide moiety at position 4 improved inhibitory activity. 

The most promising compound XZ-259, **77** in this series was taken for further development (96-98).

*Quinolone-3-carboxylic acids: The birth of second FDA-approved IN inhibitor*

Elvitegravir which was second compound well advanced in clinical trials developed jointly by Japan Tobacco and Gilead Sciences (99). Elvitegravir (84) was designed by modifying the quinolone antibiotics that do not have IN activity. In their original design, the Japan Tobacco workers attempted to incorporate DKA motif into the quinolone core (Table 11). The designed structure compound 78 did not show IN inhibitory activity but its precursor derivative 79 displayed an IC_50_ of 1.6 µM. This activity proved that a monoketo moiety could be an effective replacement for the DKA motif. Further optimization including introduction of the 2-F, 3-Cl aryl substitution and hyroxyethyl group on the quinolone N1 led to a remarkable increase in potency for compounds 80 and 81. The 7-methoxy substituent and changing hydroxyethyl to a valinol derived substituent was found to be beneficial for cell-based activity. Combining the valinol group, the haloaryl substitution and the 7-methoxy group resulted in Elvitegravir with an MT-4 activity less than 1 nM (100-102). In 2012, Stribild (Genvoya^®^), which contains Elvitegravir, Cobicistat, Emtricitabine and Tenofovir was approved as the first once-daily four-drug (“quad”) pill .

Low nanomolar potencies of this series revealed that monoketo acid motif can effectively coordinate two metal ions similar to diketo acids. It has been found that Elvitegravir is mechanistically identical to the other classes of strand transfer inhibitors and exhibits cross-resistance with DKA and Raltegravir derived mutations (103-105).

After FDA-approval of Elvitegravir, many groups started modification of quinolone core of Elvitegravir. One of these efforts led to naphthyridinone-containing inhibitors which were designed by incorporation of nitrogen to the fused rings of Elvitegravir. Resultant compounds examplified by compound 85 showed low nanomolar potencies (EC_50_ = 7.6 nM) (106). 

As a part of our research program aimed at discovering new anti-HIV-1 agents, we reported some novel quinolone derivatives by integration of fragments of two IN inhibitors scaffolds. Salicylhydrazides as exemplified by the compounds 86, 87 were identified as novel IN inhibitors (ST IC_50_ = 1.1 µM), but their inherently high cytotoxicity limited their therapeutic application (107). So we merged salicylhydrazide pharmacophore with 4-quinolone-3-carboxylic acid platform. Designed compounds (for example, compound 88) showed modest anti-HIV-1 activity with no considerable cytotoxicity in cells cultures (108). 

In our recent published work, we sought to focus on modification of 4-quinolone-3-carboxylic scaffold at N-1, C-2, C-7, and C-8 positions (general structure of 89). Most of the designed compounds exhibited inhibitory effects against the single-cycle replicable HIV NL4-3 in Hela cells cultures with appropriate safety profile. SAR analysis illustrated that introduction of aryl/alkyl substituents at N-1 and C-2 positions did not improve anti-HIV-1 activity significantly, while modifications at C-7 and C-8 positions had great influence on antiviral potency. In this series compound 90 was the most promising and taken for further optimization (109, 110).

In the literature some quinolone based IN inhibitors were reported that displayed modest integrase inhibitory activities (Figure 12) (111-115).

A research group at Shionogi and GlaxoSmithKline developed a novel chelating carbamoyl pyridone scaffold with improvement of pharmacokinetic and resistance profiles. The new chemotype displayed high inhibitory potency in both enzymatic and antiviral assay and a remarkable PK profile suggestive of once daily dosing (Table 12). Using amide group on the carbamoyl pyridone core was not successful but it represented an attractive starting point for further study (116). It was sought that amide analogues involved in an undesired intramolecular hydrogen bond between the central OH and amide NH moiety which resulted in the distortion of the chelating motifs as shown in Figure 13. So next step was cyclization of the carbamoyl pyridone core in order to fix the metal chelation moiety into a desired coplanar orientation for effective metal coordination. This modification led to bicyclic compounds (for example, compound 98) with low nanomolar potency in standard antiviral assays. Introduction of an additional hydroxyl substituent on the bicyclic scaffold and subsequent cyclizitaion succeeded to deliver tricyclic carbamoyl pyridone inhibitors as potential clinical candidates. This series represented a true second generation profile in that their resistance profile distinct from those of Raltegravir and Elvitegravir (117). In this series compound 101, S/GSK1349572 demonstrated properties indicative of once-daily dosing and superior potency against resistant viral strains. Compound S/GSK1349572 khown as Dolutegravir got FDA approval for use in HIV-1 infected patients in August 2013 (118, 119).

In 2016 a group at GlaxoSmithKline developed novel series of 8-hydroxyquinoline tetracyclic lactams through incorporating structural elements from two different literature scaffolds including naphthyridinone and carbamoyl pyridone (Figure 14).

This series of IN inhibitors examplified by compound 103 had a virological profile camparable to other second generation integrase stand transfer inhibitors. However, they needed further optimization for improvements in the pharmacokinetic profiles (120). 

*Binding modes of IN inhibitors*

In spite of advantages of IN to its counterpart virally encoded enzymes RT and PR, progress against IN was very much lagging that of the other two viral enzymes. It was because of numerous reasons such as enzyme’s intractability *in vitro, *limited or misleading early structural information and early inhibitors resulting from improper assay systems. One of the main reasons could be the lack of a complete DNA bound HIV-1 IN crystal structure. Therefore, binding modes of IN inhibitors at the active site remained elusive. Although X-ray structures of individual IN domains and and their combinations have been reported, most of these structures showed differences in conformations of the active site residues and also lacked two-divalent metals bound in the active site. So there was a need for full-length IN−DNA−inhibitor complex model for a better understanding of binding mode of inhibitors (121). In 2010, Hare *et al.* showed that prototype foamy virus (PFV) integrase and HIV-1 integrase had a high level of amino acid sequence identity with a calculated RMSD of 1.04 Å. So, PFV IN can be considered as a convenient proxy for structural studies of IN inhibitors. Hare *et al.* reported crystal structure of full-length integrase from the prototype foamy virus in complex with its cognate DNA and two Mg^2+^ ions. Based on the the retroviral intasome structure an integrase tetramer tightly associates with a pair of viral DNA ends (Figure 15). Moreover, crystals were soaked in presence of both Mg^2+^ with Raltegravir (MK0518) and Elvitegravir (GS9137). The crystallographic studies of the PFV intasome with divergent IN inhibitors explained how they bind within the active site (Figure 16). It was found that after the processing of viral DNA, the active site of IN adopts an active conformation in which the carboxylate groups of Asp128, Asp185 and Glu152 (equivalent to Asp64/Asp116/Glu152 in HIV-1 IN) coordinate two Mg^2+^ ions in close proximity to the reactive 3*ꞌ*-OH of the viral DNA. One metal ion, coordinated by Asp 128 and Glu 221 activates the 3*ꞌ*-OH group of the viral DNA for strand transfer, whereas the second one, bound by Asp 128 and Asp 185 destabilizes the scissile phosphodiester group in target DNA. Once the inhibitor binds in the active site (for example MK0518 and GS9137), the chelating moiety interacts with the two Mg^2+^ by moving away the crucial 3*ꞌ*-OH end of viral DNA from the active site by more than 6 Å. The newly formed complex is then stabilized by the halobenzyl moiety by replacing the 3*ꞌ*-A17 displaced from the active site. Properly oriented halobenzyl group fits within a tight pocket formed by cytosine 16 (C16) and guanine 4 (G4) and makes π-stacking interaction with C16. These interactions lead to removal of viral 3*ꞌ*-OH from the active site, so it is not in proper orientation to attack target DNA. In this way, IN starnd transfer inhibitors render it catalytically inactive (Figure 17 and 18). These observations have underlined that both the chelating motif and the hydrophobic portion of the inhibitor are essential for IN binding (122-124).

Structural and functional analysis of Dolutegravir bound to PFV intasome illustrated that Dolutegravir binds to the active site of IN similar to other IN strand transfer selective inhibitors (Figure 19). Three coplanar oxygen atoms of Dolutegravir chelates the two Mg^2+^ ions and the difluorobenzyl group occupies the hydrophobic pocket. In comparison with Raltegravir, Dolutegravir possesses a longer and more flexible linker allowing the hydrophobic group to enter further in the pocket. This better stacking confers more van der waals interactions of hydrophobic group with IN active site. Dolutegravir demonstrated a higher genetic barrier and limited cross-resistance to Raltegravir/Elvitegravir that probably is due to different binding pattern within the active site. Thus, the next-generation INSTIs design has to contain a strategic flexible linker coupling chelating motif to the proper oriented hyrophobic region that could give the molecule the possibility of establishing favorable interactions with the IN active site (125, 126).

Different hypothesis have been presented about selectivity of IN inhibitors against strand transfer step. In 2012, Hare *et al.* reported crystal structures of PFV IN bound to unprocessed viral DNA prior to 3*ꞌ*-P and explained selectivity of known IN inhibitors. It was shown that binding of IN inhibitors to the active site in pre-3*ꞌ*-P configuration would require the displacement of the 3*ꞌ*-terminal AAT trinucleotide in contrast to the displacement of only one deoxyadenosine at the ST step. Therefore, the energetic barrier needed for such a displacement might be difficult to reach, and a compound would require to establish additional interactions with the active site in the pre-3*ꞌ*-P complex in order to balance the energetics of binding and maintain its potency (127).

*Essential pharmacophores of IN inhibitors*

Minimal HIV-1 IN pharmacophore model that utilized as a platform for inhibitor design is graphically shown in Figure 20. 

The model consists of a planar heteroatom chelating motif that binds to a pair of divalent magnesium metal centers held in place by a triad of aminoacid carboxylate residues (Asp64/Asp116/Glu152) and a flexible linker and an aromatic group extend into an adjacent hydrophobic space.A group of researchers reported some model templates for metal heteroatom chelation (Figure 21 and 22). They identified that the heteroatom triad involved in metal binding should consist of hard Lewis base donor atoms to match the hard Lewis acid character of the Mg^2+^ ions. 

It was found that the triad should possess a geometry that results in the formation of optimal chelate ring sizes, 5+6 or 6+5 membered rings that is observed in chelating moiety of Raltegravir, Elvitegravir and Dolutegravir (128).

## Conclusion

There is no doubt that, since the discovery of HIV-1 IN as a therapeutic target for HIV-1 treatment, the field of HIV-1 IN inhibitors has made remarkable progress. Inhibitors of IN are expected to possess inherent selectivity benefit since there is no human homologue. After FDA-approval of Raltegravir, IN inhibitors have gained a definitive place in the first line of treatment of HIV-1 infection. Various structural classes of IN inhibitors started with the diketo acid derivatives have been identified so far. The research in this area has led to intoduction of 3 FDA-approved IN inhibitors, Raltegravir, Elvitegravir and Dolutegravir. In this review, we covered the structural and functional properties of HIV-1 IN, developments in the field of IN inhibitors and their binding modes. 

We categorized IN inhibitors in several structural classes and provided some information on the SAR of them allowing the elaboration of new insights into the design of novel IN inhibitors.
